# The Developmental Trajectories of Children’s Reorientation to Global and Local Properties of Environmental Geometry

**DOI:** 10.1037/xge0001265

**Published:** 2022-08-04

**Authors:** Matthew G. Buckley, Luke J. Holden, Alastair D. Smith, Mark Haselgrove

**Affiliations:** 1 School of Psychology, Aston University; 2 School of Psychology, University of Nottingham; 3 School of Psychology, University of Plymouth

**Keywords:** spatial cognition, reorientation, development, geometry, navigation

## Abstract

The way in which organisms represent the shape of their environments during navigation has been debated in cognitive, comparative, and developmental psychology. While there is evidence that adult humans encode the entire boundary shape of an environment (a global-shape representation), there are also data demonstrating that organisms reorient using only segments of the boundary that signal a goal location (a local-shape representation). Developmental studies offer unique insights into this debate; however, most studies have used designs that cannot dissociate the type of boundary-shape representation that children use to guide reorientation. Thus, we examined the developmental trajectories of children’s reorientation according to local and global boundary shape. Participants aged 6–12 years were trained to find a goal hidden in one corner of a virtual arena, after which they were required to reorient in a novel test arena. From 10.5 years, children performed above chance when the test arena permitted reorientation based only on local-shape (Experiment 2), or only global-shape (Experiment 3) information. Moreover, when these responses were placed into conflict, older children reoriented with respect to global-shape information (Experiment 4). These age-related findings were not due to older children being better able to reorient in virtual environments per se: when trained and tested within the same environment (Experiment 1), children performed above chance from 6 years. Together, our results suggest (a) the ability to reorient on the basis of global- and local-shape representations develops in parallel, and (b) shape-based information is weighted to determine which representation informs reorientation.

Successful navigation requires the traveler to update their spatial location by combining information from external environmental cues with internal representations of self-movement (Gallistel, 1990). If, for any reason, the traveler becomes disoriented, a process of reorientation must take place before the journey can recommence. For example, upon exiting large buildings (e.g., shopping complexes, hospitals, and airports), or emerging from an underground transport network, people often pause to “gather their bearings” before continuing along their way. Over the last three decades, a prolific literature has established that a wide variety of animals reorient with respect to the geometric information provided by the boundaries of an environment, including ants (Wystrach & Beugnon, 2009), fish (Sovrano et al., 2002), chicks (Vallortigara et al., 1990), mountain chickadees (Gray et al., 2005), pigeons (Kelly et al., 1998), rats (McGregor et al., 2004), rhesus monkeys (Gouteux et al., 2001), as well as adult humans (Redhead & Hamilton, 2007, 2009), and children (e.g., Hermer & Spelke, 1996, 1994). However, despite the pervasive use of geometric information across species, there is considerable debate in the developmental (e.g., Newcombe, 2019), cognitive (e.g., Cheng et al., 2013; Lee, 2017), comparative (Pearce, 2009), and neuroscientific (e.g., Dudchenko, 2010; Jeffery, 2010) literature as to exactly how organisms encode information about boundary geometry during reorientation. Here, much research has focused on whether organisms encode a global representation that encompasses the entire boundary shape of an environment or, instead, encode local representations of only the segments of the boundary that signal a goal location.

A theory of reorientation, that has influenced research in multiple disciplines, suggested that organisms rely on the global shape of an environment to guide reorientation (Cheng, 1986; Gallistel, 1990; Wang & Spelke, 2002). This notion can be traced back to the seminal comparative studies conducted by Cheng (1986), who observed that rats encoded the location of food with respect to the ambiguous shape of a rectangular environment, even in the presence of landmarks that unambiguously predicted the location of the food. On the basis of these findings, Cheng proposed a geometric module for reorientation, which: (a) encodes the global shape properties of an environment, and (b) is impervious to the influence of learning about nonshape cues, such as landmarks. In keeping with the ubiquitous use of shape information for guiding reorientation across species, Gallistel (1990; see also Hermer-Vazquez et al., 1999) advocated the evolutionary benefit of relying on geometric cues within the environment, arguing that while the appearance of an environment may change over time (e.g., the appearance of a mountain range will change with the weather across the seasons), the geometric layout of an environment will not (i.e., the broad shape of the mountain range will not change with seasonal weather). Consequently, it is advantageous for organisms to rely on the geometry of their environments to guide reorientation, as this remains constant. While some proponents of the geometric module have conceded that a strict interpretation of the hypothesis does not fully explain reorientation (see reviews in Cheng, 2008; Cheng et al., 2013), the notion that organisms preferentially encode global representations of boundary information has been championed in studies of human navigation (Doeller et al., 2008; Doeller & Burgess, 2008), and continues to be a matter of theoretical importance (Buckley et al., 2021; Lee, 2017).

As with many other topics in experimental psychology, a focus on the development of human navigational systems offers fundamental insights into these questions, and proposals similar to those of the geometric module have been echoed in reports of reorientation behavior in children. Here, the boundary primacy effect has been taken as evidence that young children rely on the global-shape information provided by the boundary walls of an environment to reorient, at the expense of nonshape features (e.g., Lee & Spelke, 2010). For instance, in an experiment conducted by Hermer and Spelke (1996), children were required to find a toy that was hidden under one of four identical containers that were placed in the corners of a 4 × 6.25 ft rectangular arena, comprising three white walls and one polarizing blue wall. It was, therefore, possible for children to unambiguously learn the location of the hidden toy with respect to the blue colored wall but, instead, they were observed to rely on the ambiguous shape of the rectangular walls, searching equally often in the two geometrically equivalent corners of the environment. However, despite evidence that children prioritize boundary-shape information when reorienting, several studies suggest that this finding may be parameter-dependent. For instance, when the Hermer and Spelke (1996) task was conducted in a larger rectangular arena (8 × 12 ft), children were able to use the polarizing wall to reorient (Learmonth et al., 2001; see also Learmonth et al., 2002, 2008). Children have also been shown to reorient using a polarizing colored wall in 7 × 7 ft rhombic space (Hupbach & Nadel, 2005), and in a large octagonal space (Newcombe et al., 2010). Finally, it has also been demonstrated that children are able to reorient using a polarizing colored wall in a smaller rectangular arena (4 × 6 ft), providing they are given pretraining in which they must find a hidden object in the center of a colored wall in a different shaped (equilateral triangle) arena (Twyman et al., 2007).

Like studies with rodents and adult humans (see Buckley, Smith, & Haselgrove, 2019, for a review), the data from the developmental literature summarized above reveals a mixed pattern of results. While some studies have shown that children rely on the shape of boundaries to guide reorientation (even at the expense of more predictive landmark cues) other studies have found that children preferentially use landmarks to reorient. While observations of children prioritizing landmarks during reorientation are clearly inconsistent with the notion of boundary primacy (Lee, 2017; Lee & Spelke, 2010), the fertile literature examining reorientation in children is rather more difficult to interpret in terms of the question of whether children reorient using a global representation of environmental boundaries. The problem, here, is that previous experiments have not *required* children to encode a global representation of the shape of the environment to solve the task. That is, although searching equally often at a correct or rotationally equivalent corner of a rectangular environment has been taken as evidence for children’s reliance on a global-shape representation (e.g., Hermer & Spelke, 1994), this interpretation has not gone unchallenged. For example, it has been argued that organisms can solve reorientation tasks using only the boundary-information that is close to the goal location; known as local-shape information.

The manner in which human and nonhuman animals encode a local-shape representation of a segment of a boundary has been studied extensively in previous literature. Here, researchers have provided several alternative interpretations of why rotational errors might be observed during reorientation tasks that are conducted in symmetrical environments, without the need to invoke global-shape representations. For instance, in rodents (e.g., Pearce et al., 2004), it has been argued that animals might adopt a single-wall strategy, and encode a goal location as toward one end of just one boundary wall (e.g., in a rectangle-shaped arena the goal is located at the left end of a long wall). Reorientation based on this representation would lead the animal to the correct or rotationally equivalent corner of a rectangle-shaped area; however, there is evidence that human children (Lourenco & Cabrera, 2015) and adults (Buckley et al., 2016a) do not adopt this strategy during reorientation. Another way in which local-shape representations might be encoded was offered by view-matching theories, which suggest that organisms store a “snapshot” representation of the view of boundaries at a goal location, and then reorient by reducing the discrepancy between their current view and that stored snapshot (Cheung et al., 2008; Stürzl et al., 2008). Under this framework, rotational errors are intuitive to understand, occurring whenever the views of the correct and rotationally equivalent corner are identical. However, while view-matching theories account for some reorientation behaviors that are observed in insects (e.g., Wystrach & Beugnon, 2009), there is, again, evidence that it is not a strategy adopted by children. For example, in a study Lee and Spelke (2011), children were able to successfully reorient inside a rectangular enclosure formed by low-contrast boundary walls, but were unable to reorient when the rectangular space was marked by a high-contrast sheet laid on the ground. Such results are not consistent with the computational algorithms that are used by view-matching accounts to reduce the discrepancy between the current view and a stored view, which would predict the opposite set of results (i.e., better performance with the high-contrast cues compared with the low-contrast cues; see Cheng et al., 2013, for a review). Studies of reorientation conducted with adult humans suggest the encoding of local representations that comprise the shape properties formed by the *conjunction* of boundary walls. For example, adult humans (Lubyk et al., 2012) and children (Lee et al., 2012) have been observed to reorient on the basis of the angular information provided by two joining walls (e.g., the acute corner of a rhombus-shaped arena), and adults (Buckley et al., 2016a) also reorient using the relative lengths of conjoined walls (e.g., the corner of a rectangle where a short wall is to the left of a long wall). Crucially, reorientation based on these representations would again lead participants to search in the correct or rotationally equivalent corner of a rectangular space, and offer a plausible explanation for why rotational errors are committed in symmetrical environments.

The foregoing discussion demonstrates the difficulty in experimentally dissociating reorientation behavior that is based on either local- or global-shape representations. Both types of representation are theoretically distinct, in that a specific local-shape representation only incorporates the shape properties of a segment of a boundary (e.g., that segment that signals the goal location), whereas global-shape representations encompass boundary information that is present at multiple locations (e.g., goal and nongoal locations). However, when reorientation behavior is studied within the same-shaped environment, which has been the case in nearly all developmental studies of reorientation, the same behavioral response is expected from reorientation strategies based upon global- and local-shape representations. That being the case, it is not possible to determine whether the rotational errors observed in previous studies were the result of children relying on a global or a local representation of environmental boundaries.

To dissociate reorientation based on global versus local representations of environmental geometry, it is necessary to first train and then test participants in arenas that differ in terms of one, but not the other, of these two forms of representation. One way in which this dissociation has been achieved has been through *shape-transformation* experiments (Pearce et al., 2004), in which participants are trained and then tested inside two differently shaped arenas. For instance, in shape-transformation studies conducted with adult humans (e.g., Lew et al., 2014; see also, Buckley et al., 2016a), participants were trained to find a hidden goal in a right-angled corner of a rectangle-shaped arena, after which they were placed in a kite-shaped arena constructed from the same walls as the training arena. In these experiments, the overall shape of the training and test environments differed, and so reorientation based on a global-shape representation will not lead to preferential search in any corners of the test environment. The training and test environments do, however, share the same local-shape cues (e.g., a short wall to the left of a long wall), and adult humans (Buckley et al., 2016a; Lew et al., 2014) have been observed to preferentially search in corners of the test arena that shared the same local shape cues as the corner of the training environment that signaled the goal location.

To the best of our knowledge, only one shape-transformation experiment has been reported in the developmental reorientation literature. Here, children aged between 2 and 4 years learned the location of a photograph hidden in one of the corners of a rectangle-shaped environment, before receiving a test trial in a kite-shaped environment (Lew et al., 2014). While responses to the correct and rotationally equivalent corner were above chance in the rectangle-shaped training arena, children displayed no preference for any corner of the kite-shaped test environment. Based on these findings, it was suggested that young children do not rely on local-shape representations to guide reorientation, but instead reorient using global-shape information. It is, however, important to note that a demonstration of children failing to reorient on the basis of local geometry does not necessarily constitute evidence of children successfully reorienting on the basis of only global geometric representation. As we note above, when training and testing are conducted within the same-shaped arena, it is not possible to unambiguously determine the contribution of global-shape representations in guiding reorientation behavior, as it is confounded with behavior based upon local-shape representations. In addition, any reorientation behavior that is observed in experiments that are conducted within a single-shaped arena are also amenable to explanations based on view-matching and single-wall strategies, even if these strategies were not used by children in related paradigms (Lourenco & Cabrera, 2015). It is, then, somewhat difficult to determine the extent to which the data reported by Lew et al. (2014) support the notion that children reorient using a global representation of environmental shape because, like previous developmental studies of reorientation, the design of these experiments cannot reveal reorientation behavior that absolutely requires a representation of the global shape.

Recently, a novel reorientation task has been developed that can isolate reorientation based upon a global representation of environmental geometry. In the *perspective-transformation* paradigm (Buckley et al., 2016b) adult humans were trained to find a hidden goal (a Wi-Fi signal) on the inside of a kite-shaped arena, before receiving a test trial on the outside of the same kite-shaped arena. During this test trial, participants demonstrated a reliable preference for searching at the previously rewarded right-angled corner which, importantly, is a behavior that is difficult to explain with recourse to local-geometry representations. For example, consider a participant trained to locate a goal inside a kite-shaped arena where a long wall on the left joins a short wall on the right to form a 90° right-angled corner. When placed on the outside of the kite-shaped arena at test, the relative wall lengths at the correct corner would now be reversed from the participant’s perspective, and the angle formed by the join of the two walls is now 270 degrees instead of 90 degrees. Furthermore, when the global shape of the training and test environment differs during the perspective transformation test, but some local shape information is preserved, participants do not preferentially search at any corners of the test environment. For instance, when trained inside a kite-shaped arena, and then tested on the outside of a rectangle-shaped arena, participants searched at all corners of the rectangular space equally (Buckley et al., 2016b, Experiments 2a and 2b). This suggests that mentally transforming or rotating a local-shape representation is not sufficient to guide reorientation when people are transferred across a boundary. Were this the case, Buckley et al. (2016b) should have observed equivalent behavior when participants were transferred from the inside to the outside of a boundary, regardless of whether the training and testing shapes were the same (Experiment 1) or different (Experiment 2). Taken together, then, the pattern of data emerging from perspective transformation experiments suggests that reorientation in this paradigm is, at some point, based on a global representation of the boundary shape and, consequently, this paradigm is uniquely suited to examining whether children reorient using a global-shape representation of environmental boundaries.

In summary, following the influential proposals of the geometric module hypothesis (Cheng, 1986; Gallistel, 1990), a number of developmental studies have examined whether children encode a global representation of environmental boundaries. To date, however, the experimental designs that have been used have failed to adequately isolate reorientation behavior that relies upon a global-shape representation. It remains, therefore, unclear at what age a global-shape reorientation strategy develops. In addition, the age at which children develop the ability to successfully reorient on the basis of local-shape information is also unclear from extant data. That is, while Lew et al. (2014) demonstrated that children aged 2–4 years did not reorient based on local local-shape information, there are no data to suggest when this local-shape reorientation strategy does emerge in childhood. Based on the fact that wider spatial abilities, at least in terms of perspective taking and mental rotation, develop between the age of 5 to 10 (Newcombe, 1989; Piaget & Inhelder, 1948/2013; Snow & Strope, 1990), it can be reasoned that children’s reorientation abilities may also develop in this period. Consequently, we recruited participants aged 6–12 years in the experiments reported here. As most studies of reorientation typically recruit children from 18 months to 6 years, our samples also provided an insight into the reorientation abilities of children beyond the ages that have traditionally been examined in previous studies.

In addition to examining reorientation abilities in an age range that is not typically studied, we also examined reorientation in virtual environments instead of conventional physical spaces, to strictly control what strategies children could use to solve our tasks. In part, this decision was motivated by a curious effect pertaining to the timing of manual disorientation procedures that are used in studies of reorientation conducted in physical spaces. In a previous developmental study in which children were transferred across the boundary of a shape, two-year old infants watched an experimenter hide a toy at a corner of a rectangle-shaped box that was located in a circular enclosure, and were then required to relocate the toy after being disoriented (spun around with eyes covered) and transferred to the opposite side of the boundary (Lourenco et al., 2005). Providing the disorientation procedure occurred following the transfer across the boundary, children were successful in relocating the toy. However, children were unable to reorient if they were disoriented before being transferred across the boundary. While it is somewhat difficult to interpret the effect of moving the point at which children were disorientated during the task, it is clear that this is a variable than influences reorientation behavior. Moreover, in laboratory studies in which children have been transferred from the inside to the outside of a physical boundary (Lourenco et al., 2005; see also Huttenlocher & Vasilyeva, 2003; Lourenco & Huttenlocher, 2007), the boundary walls of the arena were small enough for participants to see over. Consequently, from the outside of the box, children could view the spatial properties of the inside of the box and, that being the case, it is again not possible to determine whether children reorient on the basis of a global or local representation of boundary shape—as they have access to both representations from any viewpoint in the environment. To combat these issues, we used virtual environments in the experiments reported here, as participants can be transported to novel locations within novel environments, at which point they must engage in a reorientation process. This transportation procedure has been successfully used in developmental studies of boundary encoding (Negen et al., 2018) and, crucially, permits an assessment of reorientation behavior without any requirement for manually disorienting children. In addition, virtual environments are not constrained by pragmatic considerations such as the amount of laboratory space available. Consequently, it was possible for us to build environments in which children could not see over the walls, while also controlling for differences in the height of participants and angle of view, as well as the potential confound of the speed of travel in younger versus older children.

The purpose of the current experiments, then, was to address long-standing gaps in our knowledge of how children use both local- and global-shape information to reorient. To achieve this, we conducted four experiments using a hidden-goal paradigm that has been used extensively in human and animal research, and which we have successfully used with children in studies that examined the developmental trajectories of navigation based on intramaze and extramaze landmarks (see Buckley et al., 2015). Participants aged between 6 to 12 years were trained to locate a hidden goal in a virtual environment, before receiving a time-limited test trial conducted without the hidden goal. During this test trial, if participants were able to successfully reorient, they should spend a large amount of time searching at the corner of arena that previously indicated the goal location.

As hidden goal tasks conducted in virtual environments are not frequently used in studies of reorientation in children, Experiment 1 was conducted to ensure that, having been trained to find a hidden goal in a right-angled corner of a kite-shaped arena, children would search in the rewarded corner during a test trial also conducted in a kite-shaped arena. Experiment 2 assessed the developmental trajectory of children’s ability to reorient using local-shape representations with a shape-transformation paradigm. Here, children were trained in a kite-shaped environment, before receiving a test trial in a rectangle-shaped environment. While it has been demonstrated that children aged 2–4 years do not reorient on the basis of local-shape information (Lew et al., 2014), the age at which children reliably *do* use local-shape information for reorientation remains an open question. In Experiments 3 and 4 we used the perspective-transformation paradigm (Buckley et al., 2016b; Buckley, Smith, & Haselgrove, 2019) to assess the developmental trajectory of reorientation with respect to global-shape representations. Here, participants were trained on the inside of a kite-shaped (Experiment 3) or a cross-shaped (Experiment 4) environment, before receiving a test trial on the outside of the same-shaped environment. As we outlined above, and have argued previously (Buckley et al., 2016b; Buckley, Holden, et al., 2019; Buckley, Smith, & Haselgrove, 2019), after training on the inside of an environment, successful navigation to the same corner on the outside of the environment requires a global representation of the boundary shape of the arena. To accurately characterize the development of reorientation using local- and global-shape representations, we performed developmental trajectory analyses in all experiments (see Thomas et al., 2009).

## Experiment 1

The purpose of Experiment 1 was to validate the use of a virtual environment to assess children’s reorientation behavior. While we have successfully used virtual environments to study the developmental trajectory of navigation based upon intra- and extramaze landmarks cues previously (Buckley et al., 2015), it remains the case that studies of reorientation with children are usually conducted in physical laboratory environments. Given this, we felt it prudent to verify that children could successfully reorient with respect to boundary shape information in a virtual world. To assess this, participants were required to find a hidden goal located in a right-angled corner of a kite-shaped virtual arena, after which they were given a test trial conducted inside of the same kite-shaped arena. Unbeknownst to participants, during this test trial the hidden goal was removed from the environment, and participants were allowed to search for 60s. If children were able to reorient with respect to the boundaries of the kite-shaped virtual environment, then during the test trial, we would expect participants to preferentially search at the right-angled corner that contained the goal location during training, compared with the alternative right-angled corner.

### Method

#### Participants

For all of the experiments reported in this article, children aged 6–12 years were recruited, and took part in only one of the experiments. G*Power 3.1.9.7 was used to determine an appropriate sample size for all experiments. Previous studies of boundary-based reorientation in children have adopted group designs, in which participants were categorized based on their age (e.g., 2-3 year-olds, 4-5 year-olds, 6-7 year-olds, etc.); thus, the extant literature could not provide an estimate of what effect size we would expect from our developmental trajectory analyses, in which age is treated as a continuous variable. Consequently, for our sample size calculations, we conservatively assumed a medium effect size, which was smaller than the effect sizes we have observed in previous studies in which we continuously related children’s age to their use of intramaze and extramaze landmark cues in a virtual environment (see Experiment 1; Buckley et al., 2015). As we were interested in whether age predicted reorientation performance in the current experiments, we then computed the required sample to detect medium-sized effects with a single predictor variable, with power of .80. This analysis revealed a required sample size of 55. Given that, a priori, we were unsure how many children would be excluded from our analysis based on failing to learn the task (see Data Analysis section), we overrecruited participants within each experiment to maintain power following any data that was not included in our analyses (which turned out to be minimal; see Results sections).

For Experiment 1, 69 children (31 females, 38 males), with a mean age of 8.81 years (*SD* = 1.57), were recruited during Summer Scientist Week, an annual public engagement event conducted at the University of Nottingham (for more details, see https://www.nottingham.ac.uk/psychology/outreach/summer-scientist-week/summer-scientist-week.aspx). All children had normal or corrected-to-normal vision, and participated with full parental consent. The ages of male (*M =* 8.68, *SD =* 1.64) and female (*M* = 8.89, *SD* = 1.50) participants did not differ, *t*(66) = .55, *p* = .58. In return for participation, all children were given a token that allowed them to play a game at the event. All studies in this article received ethical approval from the School of Psychology Ethics Committee, University of Nottingham.

#### Material

**Figure 1 fig1:**
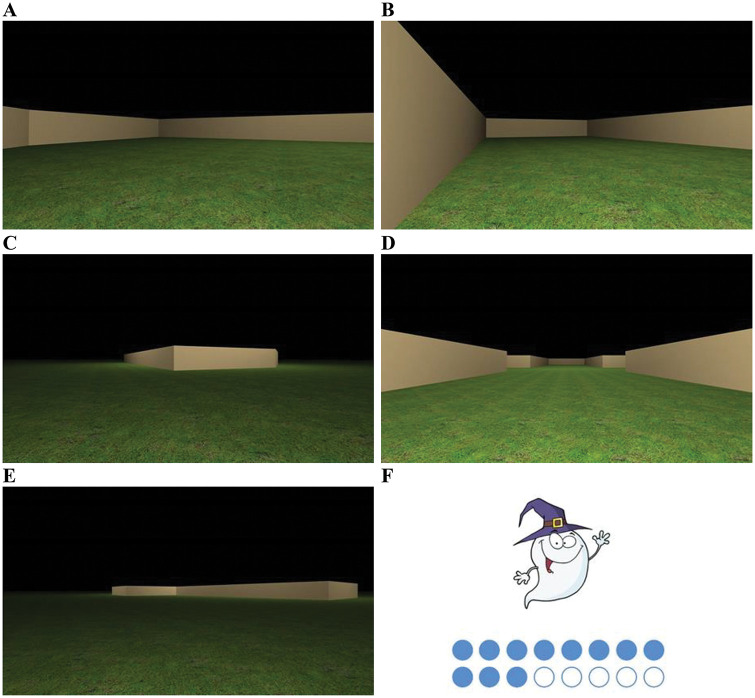
Screenshots of the Reorientation Task Used in Experiments 1–4, Which Was Framed as a “Ghost-Hunter” Computer Game *Note*. (A) The inside of the kite-shaped arena that was used for training in Experiments 1–3, and at test in Experiment 1. (B) The inside of the rectangle-shaped arena that was used at test in Experiment 2. (C) The outside of the kite-shaped arena that was used at test in Experiment 3. (D and E) The inside and outside of the cross-shaped arena that was used for training and test, respectively, in Experiment 4. (F) The screen displayed to participants when they had found the goal location on each training trial, with the trial counter that allowed participants to track their progress through the “game.” See the online article for the color version of this figure.

All virtual environments were constructed, compiled, and displayed using MazeSuite software (Ayaz et al., 2008; see http://www.mazesuite.com). The virtual environments, which participants viewed from a first-person perspective with a field of view of 45°, were displayed on a Microsoft Windows partition of an Apple Mackintosh model A1224 (EMC2133) computer with a screen of 27.40 × 43.40 cm. While conducting the experiment, a small table (approximately 60 cm in height) and accompanying chair were used to ensure the computer screen was at the child’s eye height. Two virtual arenas were used in Experiment 1: (a) a square instructional arena, and (b) a kite-shaped training and test arena. The instructional arena was used to demonstrate the task requirements to participants (see Procedure), during which we wanted to highlight that cues in the environment could be used to learn the goal location. However, to keep participants naïve to the solution of the experimental task, we did not want to instruct participants to use the shape of the environment when demonstrating the task. Consequently, we used a geometrically ambiguous square arena, and colored walls provided unambiguous cues to learn the goal location in the instructional arena. Here, we colored two adjacent walls cream (using the 0–255 RGB scale used by Mazesuite this was defined as: 204, 178, and 127), and the remaining two walls were blue (RGB: 78, 204, and 229). For the kite-shaped arena, all walls were colored cream. A grass texture was applied to the floor of each arena and a uniform black texture was applied to the ceiling (see Panel A of Figure 1). Here, and elsewhere, we define the virtual size of these arenas by assuming a walking speed of 2 m/s. Thus, the perimeter of the square arena was 72 m (each wall: 18 m). The perimeter of the kite-shaped arena was also 72 m, with the small walls being 9 m in length and the long walls 27 m. The kite was configured such that it contained two right-angled corners with the remaining two angles being 143.14° and 36.86°. The height of both arenas was approximately 2.5 m. The goals within the arenas were square-shaped regions (2 m × 2 m, invisible to participants) that were always placed such that two sides of the goal region touched the short and long wall of the kite.[Fig fig1]

#### Design and Procedure

This section will first describe the instructions that were provided to participants, before outlining the procedural details of the experiment.

##### Instructions

Before beginning the experiment, the experimenter gave standardized verbal instructions to all participants, combined with a brief demonstration of the experimental task. Here, we outline the verbal instructions in italics, and describe the demonstration in standard font. At the beginning of the experiment, the following instructions were recited to participants:

*The aim of this game is to find Gabby the Ghost. Gabby is hiding somewhere in a room, but we cannot see her. So, what we have to do is walk around the room, and if we bump into Gabby, she’ll appear on the screen to let us know we found her. Now, the trick to the game is to remember where Gabby is hiding, because she always hides in the same place. So, I’ll show you how to play the game first, and then we’ll let you have a go. Is that ok?*

The experimenter then demonstrated two trials of the experiment in the square instruction-arena. At the beginning of the first demonstration trial, the experimenter began in the center of the arena, and rotated the scene through 360° and talked through the layout of the arena. *So, this is what the game looks like. You can see we are in a big room* [experimenter rotates the view to show the participant the whole arena], *and somewhere in here is Gabby, but we cannot see her. Now, on the first go we just have to walk around and hope we find her, because we do not know where she is yet.* [The experimenter would then take a meandering path around the arena ending at the corner of the arena where the two blue walls intersect]. *She appears on the screen when we run into her though – just like that.* The first instruction trial would last approximately 30 s and, at the beginning of the second trial, the experimenter again explained that Gabby would always hide in the same place: *So, the trick of the game is to remember where Gabby is hiding, because she’s in the same place on every go. I think she was in the blue corner last time, so I’m going to go straight back there* [the experimenter then walked in a direct route to the hidden goal], *and there she is.* The participant was then given an opportunity to find Gabby on one trial in the instruction arena. *Do you want to have a go? Ok, so you move around using the arrow keys like I did. These arrows will move you forward and backward, and these two will turn you around* [said while the experimenter pointed to the cursor keys in turn].

Once participants had completed this practice trial, they progressed onto the training trials within the kite-shaped arena of the experiment proper. Participants were told: *Level two of the game is loading*. *In this level it’s a little harder to find Gabby, because all the walls are the same color. We cannot just go to the blue corner now, so we’ll have to work out another way to tell where Gabby is hiding. Ready?* If the time during any training trial of the experiment reached 55 s, the experimenter explained: *Now, if we do not find Gabby after a while, she puts up a white flag to show us where she’s hiding. So if you see a flag, that’s Gabby. Try to remember where she is for the next go though, so we can find her without any help.* Once participants had completed the training trials of the experiment they were given a final test trial. Before the test trial, participants were told*: In this level Gabby is really hard to bump in to, because she’s curled up into a tiny ball. She’s still hiding in the same place as before though, so we just have to go back to where we’ve always found her.*

##### Experiment

Participants sat not more than 50 cm from the screen. Presses on the “up” and “down” cursor keys permitted the participant to move forward and backward within the arena, respectively. Presses on the “left” and “right” cursor keys permitted the participant to rotate counterclockwise and clockwise within the arena, respectively. Once the practice trial within the square instruction-arena was completed, participants received 16 training trials before receiving a single test-trial.

**Figure 2 fig2:**
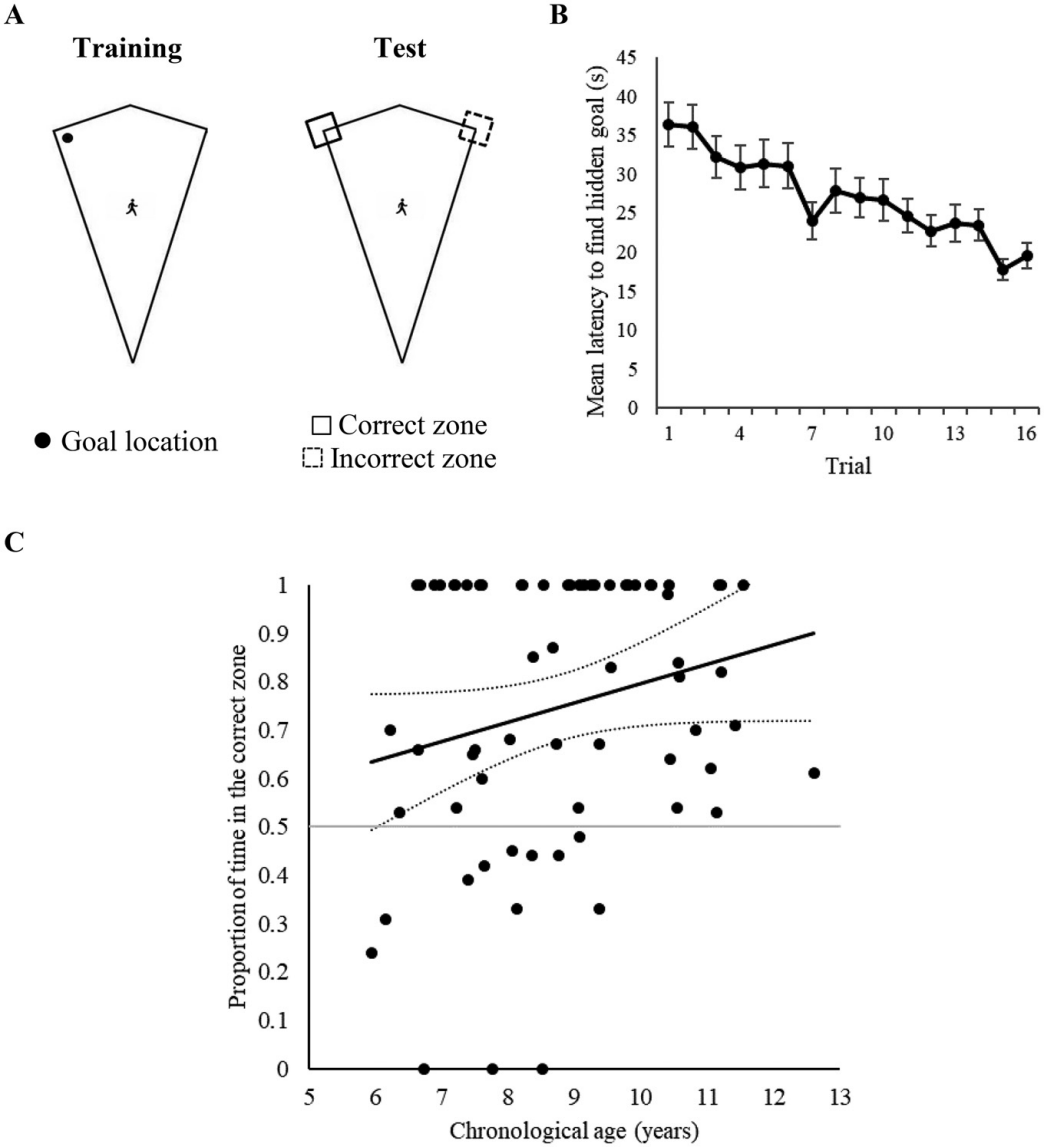
Design and Data From Experiment 1 *Note*. (A) Schematic diagrams of the training and test environments. The location of the person indicates whether children were navigating on the inside or outside of the environment. (B) Mean latencies to find the goal during training, collapsed across all participant ages. Error bars represent ±1 *SEM*. (C) Proportion of time spent in the correct zone at test, plotted by individual ages. The solid black line represents the linear regression model of age predicting proportion scores, and the dotted lines represent the upper and lower 95% confidence intervals of the model. The solid gray line indicates chance performance at test.

On each training trial, participants always began at a point halfway between the acute and obtuse corners of the kite-shaped arena, with the direction in which participants faced randomized (between 0 and 359°) at the beginning of each trial. During training, participants were allowed to search for the hidden goal for up to 60 s on each trial, after which a flag appeared at the goal location. Training trials ended only when participants had navigated to the hidden goal. Once the hidden goal was found, participants could no longer move, a picture of Gabby the Ghost appeared on screen for 2 s, and a trial-counter incremented to allow children to track their progress through the “game” (see Panel F of Figure 1). The next training trial commenced automatically. For 35 participants, the hidden goal was located in the right-angled corner where a short wall was to the right of a long wall, and for the remaining 34 participants the goal was located where a long wall was to the right of a short wall. After 16 training trials, participants received a single 60 s test trial in which, unbeknownst to them, the hidden goal was removed from the arena. In keeping with our studies conducted with adults (e.g., Buckley et al., 2016b; Buckley, Holden, et al., 2019; Buckley, Smith, & Haselgrove, 2019), performance during this test trial was analyzed by measuring navigation within 6.48 m × 6.48 m square search zones, which were centered on all points where a long and short wall met to create a right-angled corner. These search zones were orientated such that two of their edges were parallel to a long wall of an arena, and the remaining two edges ran parallel to a short wall of an arena. As participants could only explore the inside of the arena at test, however, these search zones were functionally 3.24 m × 3.24 m square search zones (see Panel A of Figure 2). Assessing spatial behavior during extinction tests (where no hidden goal is present) is common in experiments conducted with both animals (e.g., McGregor et al., 2009) and human adults (Buckley, Holden, et al., 2019; Holden et al., 2021; Redhead & Hamilton, 2009), and we have successfully used test trials without the presence of a hidden goal in previous navigational experiments with children (Buckley et al., 2015). In keeping with the training trials, participants began the test trial at a point halfway between the apex and obtuse corners of the kite-shaped arena, and the direction in which participants faced was again randomized between participants (between 0 and 359°).[Fig fig2]

#### Data Analysis

An alpha-level of .05 was adopted for assessing statistical significance. Before analysis, data from the training phase of the experiment were screened to identify children that had failed to learn the location of the hidden goal, or otherwise disengaged with the task. Here, we applied a criterion that the last two training trials of the experiment must have been completed within 120 s. If children took longer than 120 s to complete the final two trials, it would likely indicate that they were relying on the goal location being shown to them after 60 s (see Procedure) in both of the last two trials of training, or were not immediately navigating to the goal location when it became visible.

During training, we recorded the time taken for participants to find the hidden goal, as well as their distance traveled. Similarly, during test, we recorded time spent and distance traversed within the search zones located at the correct and incorrect corner (see Procedure). While these measures are highly correlated, it is possible that they may reveal subtle differences in performance, because the time measure incorporates all behavior during a trial, whereas the path length measure only incorporates movement across the floor of the environment. For instance, during training, if there were age-related differences in time taken to find the goal, but not in distances traveled, this may reflect a difference in deciding which part of the arena to navigate to (see Buckley et al., 2015). To analyze latencies and path lengths to find the goal during training, we conducted analysis of covariance (ANCOVA), with trials (1–16) as a within-subjects factor, and age as a covariate. As we have noted previously (Buckley et al., 2015), it is necessary to mean-center the age covariate when performing these analyses, as it has been demonstrated that tests of within-subjects main effects are altered when the mean of a covariate differs from zero (see Delaney & Maxwell, 1981; Thomas et al., 2009). By mean centering age (subtracting the mean age of the entire sample from individual ages) the mean of the covariate becomes zero but, importantly, this rescaling does not influence tests of the main effect of, or interactions with, the covariate. In addition to significance values, we also report partial eta-squared effect sizes with appropriate confidence intervals (CIs; Lakens, 2013).

To analyze the test data, following Buckley et al. (2016b), we expressed the time spent and distance traveled in the correct zone as a function of the time spent and distance traveled in both the correct and incorrect zones. The calculation yielded scores that indicate the proportion of time children spent in the correct zone at test, with values closer to 1 indicating more correct search performance, and chance performance indicated by a value of .5 (i.e., no preference for the correct or incorrect zone). This measure is more informative than an analysis of raw time and path length data—as it permits us to assess if participants spent more time in the correct zone than would be expected by chance (i.e., it is not possible to determine a priori how much time a participant should spend, or how far they should travel, in the correct zone for behavior to be deemed above chance). Moreover, the definition of chance we have adopted provides the most sensitive assessment of whether participants preferentially explored the correct zone over the incorrect zone. For instance, it would be possible to define chance by expressing time in the correct zone as a function of the entire navigable space at test, but this measure would only be sensitive if it was assumed that participants would explore the entire test environment equally. However, given that participants were trained to find the goal in the corner of training environment, it should be expected that search at test will be biased toward corners of the arena. Consequently, it is much more appropriate to analyze whether participants preferentially favored searching in the (correct) corner where the goal was previously located, relative to another (incorrect) corner that shares similar geometric properties. To examine whether test performance was related to the age of participants, individual ages were regressed onto proportion scores. Following Thomas et al. (2009; see also Buckley et al., 2015), we rescaled the age predictor to reflect the months from the youngest age tested within our sample. Rescaling ages in the manner does not alter the predictive ability of age, but does adjust the *y*-intercept of the regression model such that it occurs at the youngest age within our sample.

In addition to the analyses above, we also performed analyses to examine sex differences in our samples, based upon biological sex (as assigned at birth) reported by parents at recruitment. Reliable sex differences have been observed in spatial learning experiments conducted with adults (e.g., Buckley & Bast, 2018), and recent meta-analyses have begun to determine the factors that may lead to the observation of sex differences in spatial tasks (Lauer et al., 2019; Nazareth et al., 2019). Sex differences are generally not observed when children’s reorientation abilities are examined; however, there is at least one study in which young males placed greater weight on the shape of the surrounding space, over landmark cues, compared with young females (Lourenco et al., 2011). Consequently, we performed between-subjects *t* tests on the proportion of time and distances traveled within the correct and incorrect zones during test. For training data, we averaged the time and distance traveled to find the goal across all training trials and, again, analyzed for the presence of sex differences using between-subjects *t* tests. We note here that our experiments were not designed to address the issue of sex differences, and we did not recruit samples with these analyses in mind (see Sample Size Calculations). Consequently, these analyses should be seen as exploratory in nature.

#### Transparency and Openness

For each experiment reported here, we detail how we determined our sample size, processes for identifying any data to be excluded, any data exclusions, all manipulations, and all measures in the study. Data were analyzed using options available in IBM SPSS Statistics Version 26 (i.e., no custom analysis code). The design and analysis of experiments were based on previously published manuscripts, but were not preregistered.

### Results

Preliminary screening of training data (see Data Analysis section) revealed that one 9-year male and one 9-year old female participant took longer than 120 s to complete the final two trials of the training phase and, consequently, they were removed from the following analyses.

#### Training

Panel B of Figure 2 shows that the mean latencies for participants to find the hidden goal decreased as training progressed, indicating that children learned the task. A one-way ANCOVA with a within-subjects factor of trial (1–16), and a covariate of age, revealed a significant effect of trial, *F*(15, 975) = 6.12, *p* < .001, η_p_^2^ = .09, 95% CI [.05, .10], a significant age covariate, *F*(1, 65) = 28.04, *p* < .001, η_p_^2^ = .30, 95% CI [.15, .43], but no interaction between trial and age, *F*(15, 975) = 1.47, *p* = .11, η_p_^2^ = .02, 95% CI [.00, .02]. The same ANCOVA analysis revealed a similar pattern of data for distances traveled to find the goal during training, with there being a significant effect of trial, *F*(15, 975) = 5.08, *p* < .001, η_p_^2^ = .07, 95% CI [.04, .09], a significant age covariate, *F*(1, 65) = 16.45, *p* < .001, η_p_^2^ = .20, 95% CI [.07, .33], but no interaction between trial and age, *F*(15, 975) = 1.08, *p* = .37, η_p_^2^ = 02, 95% CI [.00, .02].

#### Test

To assess whether test performance was predicted by participant age, individual ages were regressed onto proportion of time spent and distances traversed in the correct zone. As shown in Table 1, age was not a reliable predictor of performance at test, and did not account for a significant proportion of variance within the time, *R*^2^ = .05, *F*(1, 65) = 3.35, *p* = .07 or path length data *R*^2^ = .02, *F*(1, 65) = 1.61, *p* = .21. Panel C of Figure 2 presents individual time proportion scores against the ages of participants, and suggests that the vast majority of participants performed at a level that was above chance level. Moreover, by examining the point at which the lower bound confidence interval passes chance performance, it is clear that from the age of 6 years, participants were likely to perform at an above chance level.[Table tbl1]

#### Sex Differences

Between-subjects *t* tests conducted on individual latencies to find the goal during training revealed that male participants were significantly quicker than female participants (males: *M* = 24.97, *SD* = 9.17; females: *M* = 29.94, *SD* = 11.09), *t*(65) = 2.01, *p* = .049, *d* = .49, 95% CI [.002, .98]. Similarly, males spent a significantly greater proportion of time in the correct zone at test than females (males: *M* = .82, *SD* = .21; females: *M* = .66, *SD* = .34) *t*(65) = 2.43, *p* = .02, *d* = .60, 95% CI [.10, .1.09].

For path length data, between-subjects *t* tests revealed no significant differences in distances traversed to find the goal (males: *M* = 30.80, *SD* = 8.82; females: *M* = 32.47, *SD* = 12.03, *t*(65) = .65, *p* = .52, *d* = .16, 95% CI [−.32, .64], or proportion of distance traversed within the correct zone at test (males: *M* = .78, *SD* = .23; females: *M* = .66, *SD* = .33), *t*(65) = 1.73, *p* = .09, *d* = .43, 95% CI [−.06, .91].

### Discussion

The purpose of Experiment 1 was to validate the use of a virtual environment to assess children’s reorientation behavior. Children were trained to find a hidden goal in a right-angled corner of a kite-shaped area, before receiving a test-trial conducted in the same-shaped arena, but in the absence of the hidden goal. Regression analyses revealed that the age of participants did not significantly predict the proportion of time spent (or distance traversed) at the correct corner at test. Moreover, the confidence intervals around the regression model for time indicated that, from the age of 6 years, participant performance was above chance. Consequently, these data indicate that children were able to successfully reorient in our virtual paradigm.

## Experiment 2

Shape-transformation paradigms have revealed that rodents (Pearce et al., 2004), chicks (Tommasi & Polli, 2004), and adult humans (Buckley et al., 2016a, 2016b) can reorient on the basis of local-shape cues. For instance, following training to find a hidden goal in a rectangle-shaped arena where a short wall is to the left of a long wall, a test trial conducted in a kite-shaped arena will reveal a bias toward searching in the right-angled corner of the kite that shares the same local-shape cues that that were associated with the goal location in the rectangle. Given that the global geometry of the training and test environments differ in a shape-transformation paradigm, it has been argued that any preferential search behavior in the test environment must be based upon local-geometry representations (Buckley et al., 2016a, 2016b; Pearce et al., 2004). Children aged 2–4 years fail to reorient on the basis of local-shape cues (Lew et al., 2014), suggesting that reorientation based upon local-shape information is a navigational strategy that develops beyond the preschool years. The purpose of Experiment 2, therefore, was to characterize the developmental trajectory of reorientation with respect to local-shape cues in school-age children. To achieve this, we performed a shape-transformation experiment in which children were required to find the location of a hidden goal in a kite-shaped arena, before receiving a test-trial inside a rectangle-shaped arena.

### Method

#### Participants

Seventy-two children (34 females, 38 males), with a mean age of 8.97 years (*SD* = 1.67) were recruited at the same annual public engagement event as in Experiment 1. The ages of male (*M =* 8.64, *SD =* 1.73) and female (*M* = 9.33, *SD =* 1.56) participants did not differ, *t*(70) = 1.75, *p* = .09. In keeping with Experiment 1, all participants were given a token in return for completing the experiment, which allowed them to play a game at the event.

#### Material

All details concerning the materials for the square-shaped instructional and kite-shaped training arenas were the same as for Experiment 1. For the test trial in Experiment 2, a rectangle-shaped arena constructed of four right-angled corners was used (see Panel B of Figure 1). Like the kite-shaped training arena, it had a perimeter of 72 m (small walls: 9 m, long walls: 27 m), and the height of the walls was again approximately 2.5 m.

#### Design and Procedure

##### Instructions

The verbal instructions that were given to participants during the instructional and training stages of this experiment were the same as in Experiment 1.

##### Experiment

The same procedure and design used during the instruction and training stages of the current experiment was used in Experiment 1. Thus, participants began at the center of the inside of a kite-shaped virtual arena on each trial, and were required to locate the invisible goal, Gabby the Ghost, who was hidden in a right-angled corner of the arena on each of the 16 training trials.

**Figure 3 fig3:**
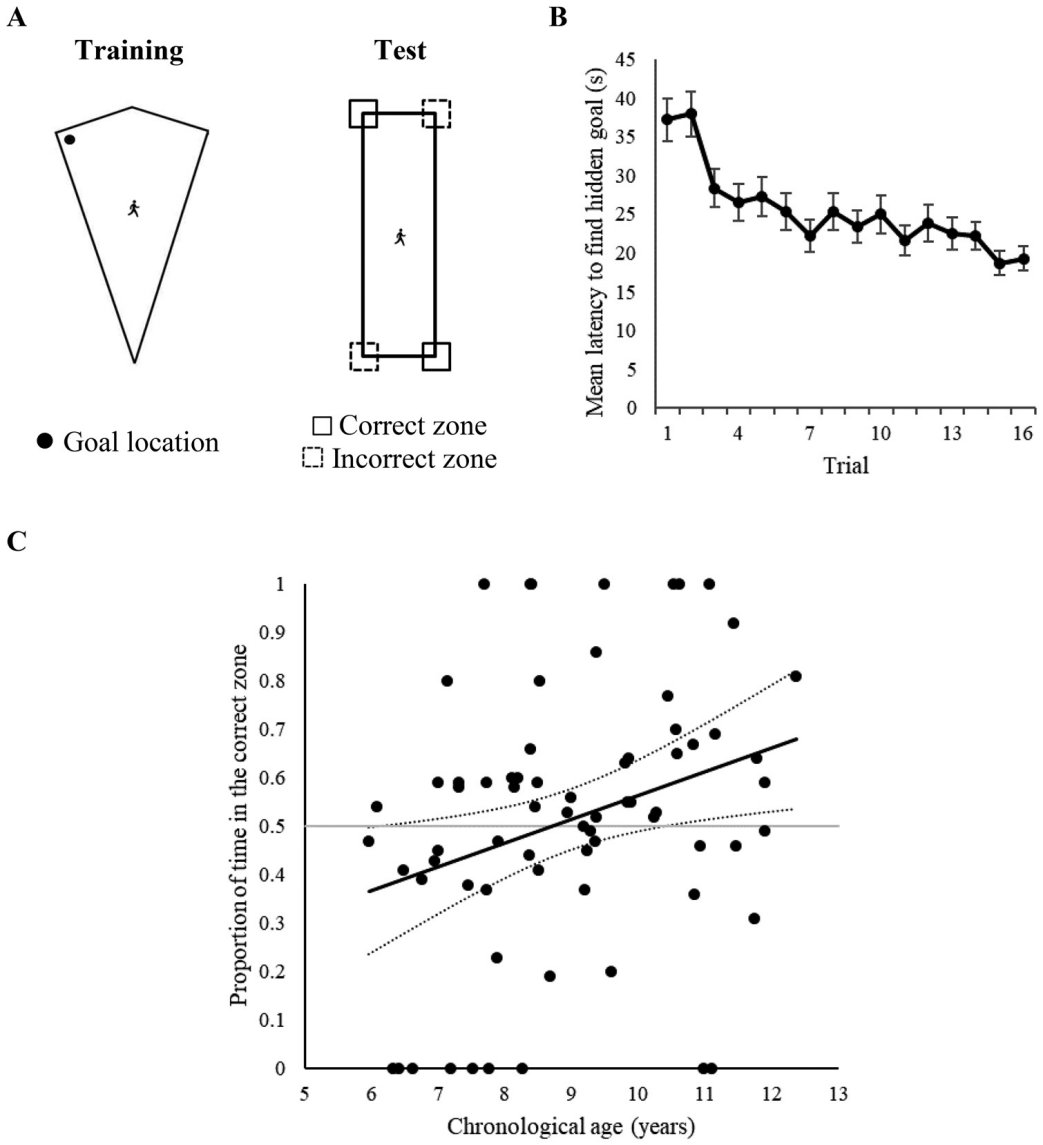
Design and Data From Experiment 2 *Note*. (A) Schematic diagrams of the training and test environments. The location of the person indicates whether children were navigating on the inside or outside of the environment. (B) Mean latencies to find the goal during training, collapsed across all participant ages. Error bars represent ±1 *SEM*. (C) Proportion of time spent in the correct zone at test, plotted by individual ages. The solid black line represents the linear regression model of age predicting proportion scores, and the dotted lines represent the upper and lower 95% confidence intervals of the model. The solid gray line indicates chance performance at test.

After 16 training trials, participants received a single 60 s test trial in which the hidden goal was removed, without the participants’ knowledge. This test was conducted inside a rectangle-shaped arena, in which participants began each trial in the center of the arena, facing a random direction. Before the test trial began, participants were verbally instructed that “*In this level Gabby is really hard to bump in to, because she’s curled up into a tiny ball. The room may look a little different this time too, but there is still a way of working out where Gabby the Ghost is hiding from us. Ready?*” As with Experiment 1 and our previous work with adults (e.g., Buckley et al., 2016b; Buckley, Holden, et al., 2019; Buckley, Smith, & Haselgrove, 2019), performance during test was analyzed by measuring the time spent and distance traveled in 6.48 × 6.48 square search zones centered on the four corners of the rectangle-shaped arena but, again, these were functionally 3.24 m × 3.24 m as participants could only search the inside of the arena (see Panel A of Figure 3). The two corners of the rectangle-shaped test arena that shared the same local-shape cues as those rewarded in the kite-shaped training arena were designated as correct zones, with the remaining corners designated as the incorrect zones.[Fig fig3]

#### Data Analysis

Data were analyzed in the same manner as described for Experiment 1.

### Results

Preliminary screening of the training data (see data analysis Experiment 1) revealed one 6-year old male participant took longer than 120 s to complete the final two trials of the training phase, and their data were removed from the following analyses.

#### Training

Panel B of Figure 3 shows that the mean latencies for participants to find the hidden goal decreased as training progressed, indicating that children learned the task. A one-way ANCOVA with a within-subjects factor of trial (1–16), and a covariate of age, revealed a significant effect of trial, *F*(15, 1035) = 8.10, *p* < .001, η_p_^2^ = .11, 95% CI [.07, .12], a significant age covariate, *F*(1, 69) = 31.07, *p* < .001, η_p_^2^ = .31, 95% CI [.16, .43], but no interaction between trial and age, *F*(15, 1035) = 1.13, *p* = .32, η_p_^2^ = .02, 95% CI [.00, .02]. The same ANCOVA used to analyze distances traversed to find the goal during training revealed a similar pattern of data, with a significant effect of trial, *F*(15, 1035) = 6.24, *p* < .001, η_p_^2^ = .08, 95% CI [.05, .10], a significant age covariate, *F*(1, 69) = 19.20, *p* < .001, η_p_^2^ = .22, 95% CI [.09, .35], but no interaction between trial and age, *F*(15, 1035) = .99, *p* = .46, η_p_^2^ = .01, 95% CI [.00, .01].

#### Test

To assess whether age predicted test performance, individual ages were regressed onto proportion of time spent and distances traversed in the correct zones. As shown in Table 1, age was a reliable predictor of performance at test, and accounted for a significant proportion of variance within both the time *R*^2^ = .09, *F*(1, 69) = 6.56, *p* = .013 and path length data *R*^2^ = .08, *F*(1, 69) = 5.88, *p* = .018. Reference to Panel C of Figure 3 suggests that as age increased, participants spent more time in the correct zones, with the lower-bound confidence interval indicating that around the age of 10.5 years, participants began to perform above chance level.

#### Sex Differences

Between-subjects *t* tests conducted on mean latencies to find the goal during training (males: *M* = 25.16, *SD* = 11.09; females: *M* = 25.74, *SD* = 10.80), *t*(69) = .22, *p* = .83, *d* = .05, 95% CI [−.41, .52], and mean proportion of time spent in the correct zone at test (males: *M* = .50, *SD* = .27; females: *M* = .53, *SD* = .29), *t*(69) = .38, *p* = .71, *d* = .09, 95% CI [−.37, .56], revealed no significant sex differences. The same pattern of data was revealed in path length data, with there being no significant differences in the mean distances traversed to find the goal during training (males: *M* = 32.60, *SD* = 12.68; females: *M* = 28.40, *SD* = 10.39), *t*(69) = 1.51, *p* = .14, *d* = .36, 95% CI [−.11, .83], or the proportion of distance traveled in the correct zone at test (males: *M* = .48, *SD* = .26; females: *M* = .52, *SD* = .27), *t*(69) = .71, *p* = .48, *d* = .17, 95% CI [−.30, .64].

### Discussion

Children in Experiment 2 completed a shape-transformation experiment, in which they were trained to find a hidden goal in a right-angled corner of kite-shaped arena, before receiving a test trial conducted in a rectangle-shaped area with no hidden goal present. Regression analyses revealed that the age of participants significantly predicted the proportion of time spent and distance traversed in the correct corner at test, and the confidence intervals around the time regression model indicated that children began to perform above chance level from the age of 10.5 years. As the global-shape of training and test environments differed, this above-chance performance in older children reflects the ability to successfully reorient on the basis of a local representation of boundary shape.

As noted previously, in their shape-transformation experiment, Lew et al. (2014) trained children aged 2–4 to find a toy hidden in the corner of a rectangle-shaped environment, before administering trials in a kite-shaped environment. While children successfully reoriented in the rectangle, there was no evidence of successful reorientation in the kite. Based on these findings, Lew et al. concluded that young children reorient on the basis of the global-shape of environmental boundaries. However, as we noted in the introduction, evidence that children failed to use local-shape cues in the kite-shaped arena provides only indirect evidence that children may have been using global-shape cues to reorient in the rectangle-shaped arena. Consequently, the purpose of Experiment 3 was to examine the developmental trajectory of reorientation based upon global shape.

## Experiment 3

The formative proposals of the geometric module (Cheng, 1986; Gallistel, 1990) inspired an abundance of reorientation studies that examined how children reorient with reference to environmental boundaries. As we noted in the introduction, while a number of developmental studies have demonstrated that, under the right circumstances, nonshape cues can be used to guide reorientation (Hupbach & Nadel, 2005; Learmonth et al., 2008; Newcombe, 2019), rather less attention has been paid to examining precisely what information children encode about environmental boundaries. That is to say, as the vast majority of reorientation experiments have conducted training and testing within the same-shaped environment, it has not been possible to determine whether children encode a representation of the global-shape of their environment, because the relative contribution of local and global shape cues in guiding reorientation behavior cannot be dissociated. The purpose of Experiment 3, therefore, was to examine reorientation behavior that is based on global-shape cues using a perspective transformation design (Buckley et al., 2016b; Buckley, Holden, et al., 2019; Holden et al., 2021), and to characterize the developmental trajectory of reorientation behavior with respect to a global representation of boundary shape. Here, participants were trained to find a goal inside a kite-shaped arena, before receiving a test trial on the outside of the same shape. Crucially, when placed on the outside of the environment, the angle formed by the conjunctions of two walls and the relationship between their lengths is reversed from training, such that local-shape representations cannot be used to guide successful reorientation behavior. As a consequence, were we to observe successful reorientation behavior, it would be based on a global-shape representation.

### Method

#### Participants

Seventy-two children (35 females, 37 males), with a mean age of 8.92 years (*SD* = 1.53) were recruited at the same annual public engagement event as in Experiment 1. The ages of male (*M =* 8.84, *SD =* 1.51) and female (*M* = 8.99, *SD* = 1.57) participants did not differ statistically, *t*(70) = .41, *p* = .68. In keeping with Experiments 1 and 2, children were given a token that allowed them to play a game at the event in return for participation.

#### Material

All details concerning the materials for the square-shaped instructional and kite-shaped training arenas were the same as for Experiment 1. For the test trial in the current experiment, participants were placed on the outside of the kite-shaped arena, where the walls were the same cream color as on the inside, and the same grass texture was applied to the floor, which extended to 780 m × 780 m. The sky was, again, rendered as a uniform black expanse. As shown in Panel C of Figure 1, the uniform grassy plain on which participants walked extended toward a black horizon.

#### Design and Procedure

##### Instructions

The same standardized verbal instructions that were given to participants during the instructional and training stages of Experiment 1 were again used. Before the final test trial, however, participants were told*: In this level Gabby is really hard to bump in to, because she’s curled up into a tiny ball. She’s also gone outside the room, so this time we’ll be on the outside of the room too. She’s still hiding in the same place as before though, so we just have to go back to where we’ve always found her. Ready?*

##### Experiment

The same procedure and design were used during the instruction and training stages of the current experiment as in Experiment 1. Having completed the training trials, participants received a test trial in which they were placed on the outside of the kite-shaped arena, with no hidden goal present. Participants began the test trial facing one of the four walls of the arena, and were located 3.15 m from the center of the wall. The four possible start locations for the test trial were counterbalanced across participants.

In keeping with our perspective transformation experiments conducted with adults (Buckley et al., 2016b), in which participants were also tested on the outside of the same kite-shaped arena as used in the present experiment, it was necessary to increase the duration of the test trial to 120 s. When on the inside of an environment (as in Experiments 1 and 2), it is possible to establish orientation based on the shape of the environment by simply rotating around the *y*-axis to bring consecutive walls into view. When on the outside of an environment, however, reorientation cannot be achieved by simply rotating around the *y*-axis. Instead, participants must travel along the *x*- and *z*-planes in order bring each wall into view and, consequently, establishing orientation when on the outside of an environment takes considerably longer compared with reorienting on the inside of an arena. To account for this, and to ensure that participants tested on the outside of an environment had sufficient time to search at the region in which they thought the hidden goal was located, the test trials in the current experiment were 60 s longer than the test trials conducted on the inside of the arena in Experiments 1 and 2.

**Figure 4 fig4:**
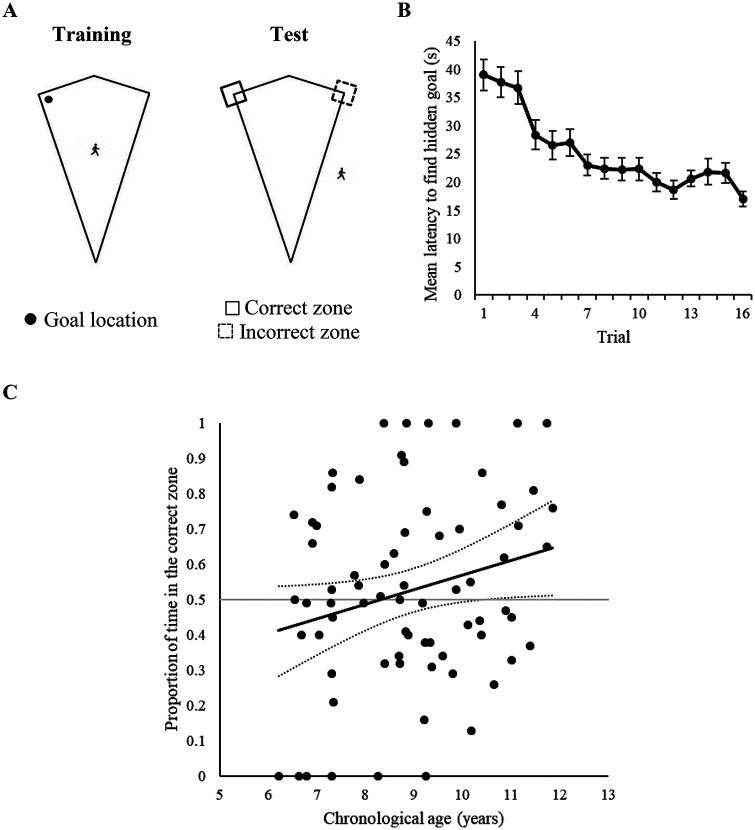
Design and Data From Experiment 3 *Note*. (A) Schematic diagrams of the training and test environments. The location of the person indicates whether children were navigating on the inside or outside of the environment. (B) Mean latencies to find the goal during training, collapsed across all participant ages. Error bars represent ±1 *SEM*. (C) Proportion of time spent in the correct zone at test, plotted by individual ages. The solid black line represents the linear regression model of age predicting proportion scores, and the dotted lines represent the upper and lower 95% confidence intervals of the model. The solid gray line indicates chance performance at test.

Also in keeping with our experiments conducted with adult participants (Buckley et al., 2016b; Buckley, Holden, et al., 2019; Buckley, Smith, & Haselgrove, 2019), behavior during the test trials was measured by recording the time spent within 6.48 m × 6.48 m square search zones, which were centered on all points where a long and short wall met to create a right angled corner. However, as participants could only explore the outside of the arena at test, these search zones were functionally L-shaped regions (long edges 6.48 m, short edges 3.24 m) that wrapped around both sides of the right-angled corners of the kite-shaped boundary walls (see Panel A of Figure 4).[Fig fig4]

#### Data Analysis

Data were analyzed in the same manner as Experiment 1.

### Results

Preliminary screening of training data (see data analysis Experiment 1) revealed that no participant took longer than 120 s to complete the final two trials of the training phase, and so all data were included in the following analyses of acquisition and test trial performance.

#### Training

Panel B of Figure 4 shows that the mean latencies for participants to find the hidden goal decreased as training progressed, indicating that children learned the task. A one-way ANCOVA with a within-subjects factor of trial (1–16), and a covariate of age, revealed a significant effect of trial, *F*(15, 1050) = 13.61, *p* < .001, η_p_^2^ = .16, 95% CI [.12, .18], a significant age covariate, *F*(1, 70) = 47.82, *p* < .001, η_p_^2^ = .41, 95% CI [.26, .52], but no interaction between trial and age, *F*(15, 1050) = 1.10, *p* = .35, η_p_^2^ = .02, 95% CI [.00, .02]. A similar ANCOVA revealed comparable results with distances traversed to find the goal in training, with a significant effect of trial, *F*(15, 1050) = 11.32, *p* < .001, η_p_^2^ = .14, 95% CI [.10, .16], a significant age covariate, *F*(1, 70) = 31.52, *p* < .001, η_p_^2^ = .31, 95% CI [.17, .43], but no interaction between trial and age, *F*(15, 1050) = 1.10, *p* = .36, η_p_^2^ = .02, 95% CI [.00, .01].

#### Test

To assess whether age predicted test performance, individual ages were regressed onto the proportion of time spent and distances traversed within the correct zone. As shown in Table 1, age was a reliable predictor of performance at test, and accounted for a significant proportion of variance within the time *R*^2^ = .06, *F*(1, 70) = 4.10, *p* = .047 and path length data *R*^2^ = .06, *F*(1, 70) = 4.66, *p* = .034. Reference to Panel C of Figure 4 suggests that as age increased, participants spent more time in the correct zone, with the lower bound confidence interval indicating that around the age of 10.5, participants began to perform at an above chance level.

#### Sex Differences

Between-subjects *t* tests revealed no significant sex differences in mean latencies to find the goal during training (males: *M* = 24.64, *SD* = 9.33; females: *M* = 25.99, *SD* = 10.89), *t*(70) = .57, *p* = .57, *d* = .13, 95% CI [−.33, .60], or mean proportions of time spent in the correct zone at test (males: *M* = .56, *SD* = .24; females: *M* = .49, *SD* = .30), *t*(70) = 1.11, *p* = .27, *d* = .26, 95% CI [−.20, .72]. The same pattern of performance was revealed in path length data, with there being no significant differences in the mean distances traversed to find the goal during training (males: *M* = 29.26, *SD* = 11.08; females: *M* = 28.26, *SD* = 12.33), *t*(70) = .62, *p* = .54, *d* = .15, 95% CI [−.32, .61], or the proportion of distance traveled in the correct zone at test (males: *M* = .57, *SD* = .22; females: *M* = .49, *SD* = .28), *t*(70) = 1.48, *p* = .14, *d* = .35, 95% CI [−.12, .82].

### Discussion

In Experiment 3, children completed a perspective-transformation paradigm, in which they were trained to find a hidden goal that was located in a right-angled corner of a kite-shaped arena, before receiving a test trial conducted without a hidden goal present, on the outside of the same-shaped arena. Having been transferred from navigating on the inside to the outside of the boundary shape, any local-shape information (e.g., a long wall to the left of a short wall) learned during training cannot be used to successfully reorient. Regression analysis revealed that the proportion of time spent (and distance traversed) at the correct corner at test was significantly predicted by age, and the confidence intervals around the time regression model indicated that children’s ability to reorient on the basis of a global-shape representation at an above chance level emerges around the age of 10.5 years.

It is worth emphasizing, at this point, that the conditions of training in Experiment 3 precisely matched the conditions of training used in Experiments 1 and 2. In all cases, participants at the same public-engagement event were required to find a hidden goal within a kite-shaped virtual environment, and the difference between these experiments was solely in the conditions of testing. Consequently, a cross-experiment comparison of the developmental trajectories of reorientation based upon either local-shape information alone (Experiment 2), or on global-shape information alone (Experiment 3) is appropriate, and suggests that both strategies appear to reliably emerge at a similar age. The implication of these results is that the developmental trajectories of reorientation based upon local and global representations of environmental boundaries are equivalent—an issue we shall return to in the general discussion. What cannot be determined from the results of Experiments 2 and 3, however, is the extent to which children of different ages will rely on local- or global-shape representations for reorientation, when both types of representation could feasibly guide behavior. Experiment 4 was conducted to address this question.

## Experiment 4

In the shape-transformation paradigm used in Experiment 2, older children demonstrated an ability to reorient on the basis of local-shape cues having been trained to find a hidden goal on the inside of a kite-shaped arena, before being tested on the inside of a rectangle-shaped arena. In the perspective-transformation paradigm used in Experiment 3, older children also displayed an ability to reorient on the basis of global-shape cues, having been trained on the inside of a kite-shaped arena, before being tested on the outside of the same kite-shaped arena. From these experiments, however, it is not possible to determine the extent to which children rely on global- or local-shape representations when both types of representation can support reorientation. That is, in shape-transformation experiments, global-shape representations cannot be used to guide reorientation due to the change in the overall boundary-shape between training and test. Likewise, local-shape representations cannot be used to guide reorientation in perspective-transformation experiments, because the transfer from the inside to the outside of the boundary shape between training and test reverses the spatial relationship between the relative wall lengths and changes the angular information of the local-shape cue that signaled a goal location during training.

**Figure 5 fig5:**
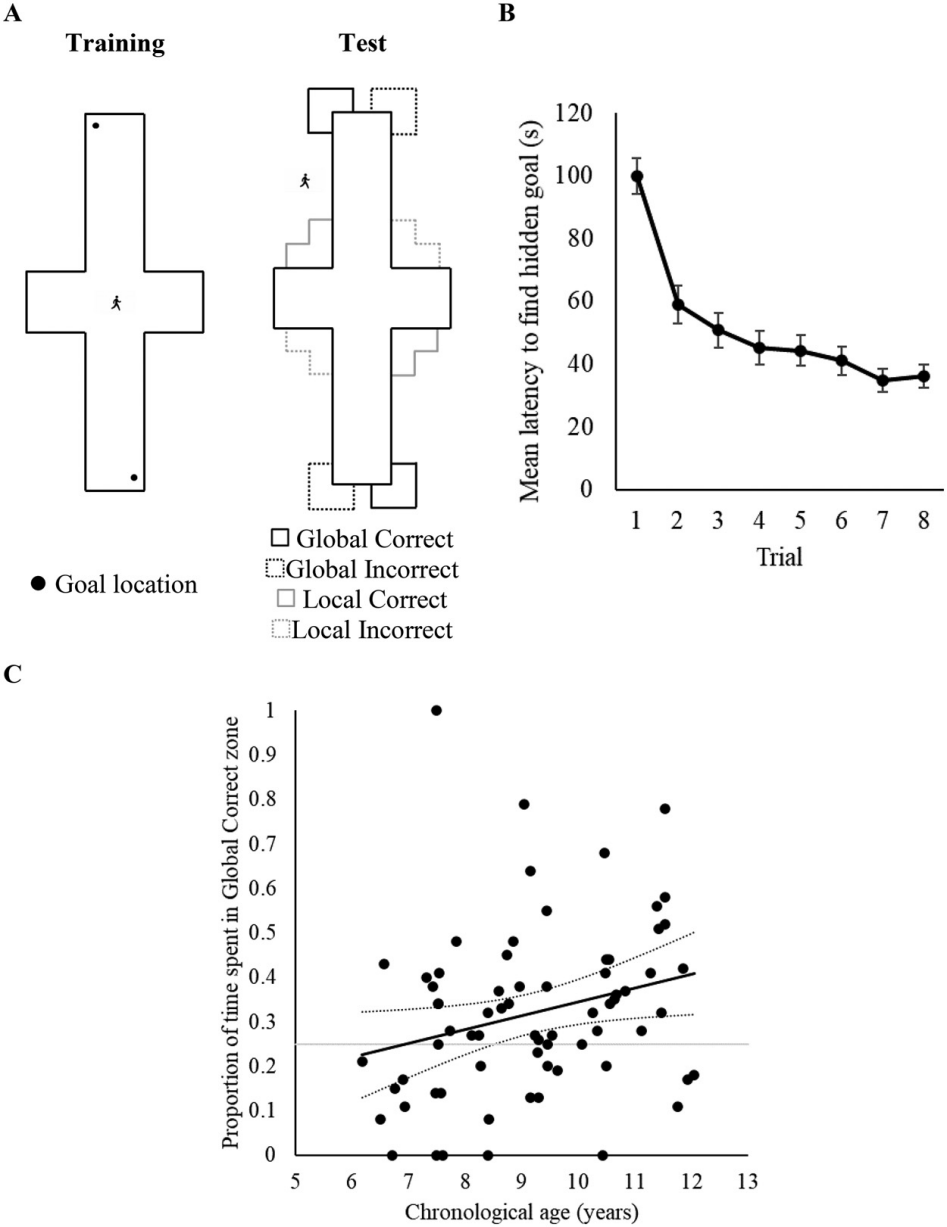
Design and Data From Experiment 4 *Note*. (A) Schematic diagrams of the training and test environments. The location of the person indicates whether children were navigating on the inside or outside of the environment. (B) Mean latencies to find the goal during training, collapsed across all participant ages. Error bars represent ±1 *SEM*. (C) Proportion of time spent in the Global Correct zone at test, plotted by individual ages. The solid black line represents the linear regression model of age predicting proportion scores, and the dotted lines represent the upper and lower 95% confidence intervals of the model. The solid gray line indicates chance performance at test.

To determine whether human adults preferentially reorient with respect to global- or local-shape representations, perspective transformation experiments have been conducted with a cross-shaped environment (see Panel A of Figure 5). Here, participants were trained to find a hidden goal in, say, a 90° corner where a long wall was to the left of a short wall. When placed on the outside of this cross-shape at test, this same local-shape cue was still present, and we refer to the area adjacent to this cue as the “Local Correct zone.” Crucially, however, the Local Correct zone is in a different spatial location to the right-angled 270° corner that is immediately outside the 90° corner that contained the hidden goal during training (i.e., on the other side of the boundary wall). We refer to the area that is adjacent to this corner the “Global Correct zone.” Consequently, at test, reorientation behavior based on a local-shape representation can be placed into conflict with reorientation behavior based on a global-shape representation. Across a series of experiments using this cross-maze arena (that have varied the number of training trials, and the start locations during training) adult humans have demonstrated a consistent preference for reorienting on the basis of a global-shape representation, over local shape-representations, by searching for the hidden goal in the Global Correct zone significantly longer than in the Local Correct zone (Buckley, Holden, et al., 2019; Holden et al., 2021). The purpose of Experiment 4 was to determine if children would display a similar preference when tested in the same paradigm.[Fig fig5]

### Method

#### Participants

Seventy children (29 females, 41 males), with a mean age of 9.07 years (*SD* = 1.60) were recruited at the same annual public engagement event as Experiments 1 to 3. The ages of male (*M =* 9.19, *SD =* 1.57) and female (*M* = 8.90, *SD* = 1.66) participants did not differ statistically, *t*(68) = .73, *p* = .47. As with all previous experiments, participants were given a token that allowed them to play a game at the event in return for participation.

#### Material

The walls of the cross-shaped environment were the same cream color as the walls used in Experiments 1–3 (long walls: 22.5 m, short walls: 9 m long, height: approximately 2.5 m). Concave corners on the inside and the outside of the cross-shaped arena comprised angles of 90°, and convex corners on the inside and the outside of the arena comprised angles of 270°. In keeping with the materials used by Buckley, Holden, et al. (2019; see also Holden et al., 2021) the hidden goals within the arenas were square-shaped regions (1.08 m × 1.08 m) that were always placed 2.48 m away from the walls of the arena, along on a notional line that bisected the corner (see Panels D and E of Figure 1).

#### Design and Procedure

##### Instructions

The same instructions were used in Experiment 4 as were used in Experiment 3.

##### Experiment

Previous studies (Buckley, Holden, et al., 2019) have revealed that adult participants rapidly learn this task. Indeed, Holden et al. (2021) have shown that participants will successfully transfer their search behavior to the appropriate global search zones at test following as few as two training trials on the inside of the cross-shaped arena. Consequently, to reduce any potential fatigue effects in our younger participants the number of training trials were reduced from our previously reported 16 to 8. Otherwise, procedural details for the eight acquisition trials were identical to Experiment 3, save for the starting location for each trial, the counterbalancing of the goal location, and the amount of time before the goal location was revealed. Participants began each trial from one of four positions, located halfway along one of the arms of the cross-shaped environment. The order of the starting positions was pseudorandomized for each participant, with the stipulation that each of the four start locations was used twice during the eight acquisition trials, and that consecutive trials never began from the same start location. The direction in which participants began facing was randomized between 0 to 359° for every trial. The hidden goal was located at either a concave or convex corner where a short wall met a long wall, meaning four possible goal locations were counterbalanced across participants. As with Experiments 1–3, we wanted to ensure that visits to the correct corner of the cross-shaped environment always resulted in finding the hidden goal and, as the cross-shaped arena contained two corners that shared the same shape properties, it was necessary for each training environment to contain two hidden goals. Finally, as the cross-shaped environment was significantly larger than the kite-and rectangle-shaped environments used in our previous experiments, the goal location was only revealed to participants after 120 s (twice the amount of time relative to Experiments 1–3).

Following training, participants received the same pretest instructions as Experiment 3, and pressing enter began a test trial conducted on the outside of the cross-shaped environment. Again, as the cross-shaped environment used in the current experiment was much larger than the environments used in Experiments 1–3, we doubled the length of this trial to ensure that participants had sufficient time to search for the absent hidden goal. Consequently, participants were given a 240 s test trial on the outside of the environment, which is in keeping with our previous studies with adult participants that have used the cross-shaped environment (Buckley, Holden, et al., 2019; Holden et al., 2021). Participants began the test trial facing one of the four long walls of the cross-shaped environment, and were located 9 m from the center of that long wall, along a notional line running perpendicular to the wall. There were four possible start locations for the test trial, which were counterbalanced across participants. Search behavior at test was measured using the same L-shaped search zones used in Experiment 3 (long edges 6.48 m; short edges 3.24 m). Global Correct zones were located on the outside of the corners where the hidden goal had been during training, and Global Incorrect zones were located at corners that were the mirror image of the Global Correct corner. Similarly, Local Correct zones were located at corners that shared the same local-shape information that was rewarded during training, and Local Incorrect zones were located at corners that were the mirror of the Local Correct zone corner (see Panel A of Figure 5).

#### Data Analysis

For the current experiment, we analyzed the proportion of time spent and distance traversed in the Global Correct, Local Correct, Global Incorrect, and Local Incorrect zones at test. The data for all analyses were treated in the same manner as Experiments 1–3, save for the way in which we calculated chance performance. In the current experiment, as we recorded time spent in four zones, chance was defined as .25.

Before analysis, and in keeping with Experiments 1–3, data from the learning phase were screened to identify children that had failed to learn the location of the hidden goal. For the current experiment, we applied a criterion that the last two training trials of the experiment must have been completed within 240 s. If children took longer than 240 s to complete the final two trials, it would likely indicate that they were relying on the goal location being shown to them after 120 s (see Procedure) in both of the last two trials of training, or were not immediately navigating to the goal location when it became visible.

### Results

Preliminary screening of training data (see data analysis section) revealed that no participant took longer than 240 s to complete the final two trials of the training phase, and so all data were included in the following analyses of acquisition and test trial performance.

#### Training

Panel B of Figure 5 shows that the mean latencies for participants to find the hidden goal decreased as training progressed, indicating that children learned the task. A one-way ANCOVA with a within-subjects factor of trial (1–8), and a covariate of age, revealed a significant effect of trial, *F*(7, 476) = 22.27, *p* < .001, η_p_^2^ = .25, 95% CI [.18, .29], a significant age covariate, *F*(1, 68) = 42.61, *p* < .001, η_p_^2^ = .39, 95% CI [.23, .50], but no interaction between trial and age, *F*(7, 476) = 1.72, *p* = .10, η_p_^2^ = .03, 95% CI [.00, .04]. A similar ANCOVA conducted upon the path length data revealed a signiciant effect of trial, *F*(7, 476) = 25.22, *p* < .001, η_p_^2^ = .27, 95% CI [.21, .31], a significant age covariate, *F*(1, 68) = 5.69, *p* = .02, η_p_^2^ = .08, 95% CI [.01, .19], and an interaction between trial and age, *F*(7, 476) = 2.73, *p* = .009, η_p_^2^ = .04, 95% CI [.01, .06]. Parameter estimates, generated from the ANCOVA, revealed that younger children traveled smaller distances to find the goal on Trial 1 compared with older children (*p* = .048), but older children traveled smaller distances on Trials 3 (*p* = .031) and 4 (*p* = .047). Crucially, age did not predict distance traveled on all other trials (*p* > .059), indicating all children had learned the task before the end of training.

#### Test

To assess whether age predicted time spent in zones at test, individual ages were regressed onto proportion of time spent and distance traversed in each zone. As shown in Table 2, age was a reliable predictor of the proportion of time spent searching in the Global Correct zone, and accounted for a significant proportion of variance within the data, *R*^2^ = .07, *F*(1, 68) = 4.85, *p* = .031. Reference to Panel C of Figure 5 suggests that as age increased, participants spent more time in the Global Correct zone, with the lower bound confidence interval indicating that around the age of 8.5 years, participants began to perform at an above chance level. As shown in Table 2 (see also online Supplemental Materials Figure S1), however, age did not predict the proportion of time spent in the Global Incorrect zone, *R*^2^ = .01, *F*(1, 68) = .77, *p* = .38; the Local Correct zone *R*^2^ = .02, *F*(1, 68) = 1.57, *p* = .22; or the Local Incorrect zone, *R*^2^ = .04, *F*(1, 68) = 2.64, *p* = .11.[Table tbl2]

For path length data, unlike Experiments 1–3, age did not reliably predict the proportion of distance traveled in the Global Correct zone, *R*^2^ = .04, *F*(1, 68) = 2.55, *p* = .12. Moreover, age failed to predict distance traveled in the Global Incorrect zone, *R*^2^ = .01, *F*(1, 68) = .88, *p* = .35; the Local Correct zone, *R*^2^ = .01, *F*(1, 68) = .73, *p* = .40; or the Local Incorrect zone, *R*^2^ = .02, *F*(1, 68) = 1.64, *p* = .21.

#### Sex Differences

In keeping with the training data analyzed in Experiment 1, between-subjects *t* tests conducted on mean latencies to find the goal found that males were quicker to locate the goal during training than females (males: *M* = 45.69, *SD* = 19.24; females: *M* = 59.00, *SD* = 25.27), *t*(68) = 2.51, *p* = .015, *d* =.61, 95% CI [.12, 1.09]. Unlike the test data analyzed in Experiment 1, however, there were no significant differences in the proportion of time that males and females spent in the Global Correct zone at test (males: *M* = .31, *SD* = .18; females: *M* = .33, *SD* = .22), *t*(68) = .45, *p* = .66, *d* = .11, 95% CI [−.37, .58].

Path length data was consistent with Experiments 1–3, with between-subjects *t* tests revealing no significant sex differences in mean distances traveled to find the goal during training (males: *M* = 102.16, *SD* = 43.81; females: *M* = 99.27, *SD* = 30.64), *t*(68) = .31, *p* = .76 *d* = .08, 95% CI [−.40, .55], or mean proportions of distances traveled in the Global Correct zone at test (males: *M* = .29, *SD* = .16; females: *M* = .33, *SD* = .20), *t*(68) = .72, *p* = .48 *d* = .17, 95% CI [−.30, .64].

### Discussion

Participants completed a perspective-transformation paradigm, in which they were trained to find a hidden goal on the inside of a cross-shaped arena, before receiving a test trial conducted on the outside of the arena. During the test, both the global- and local-shape cues that signaled the goal location during training were present; however, reorientation behavior based upon these representations was placed into conflict. Children, like adults (Buckley, Holden, et al., 2019; Holden et al., 2021), demonstrated a preference for searching at regions that were compatible with a global, rather than a local, representation of the shape of the environment, and regression analyses revealed that age only predicted the proportion of time spent searching in the Global Correct zone. Furthermore, confidence intervals indicated that children’s ability to reorient on the basis of a global-shape cue emerged around the age of 8.5 years.

In Experiments 1–3, our analyses of distance traveled within each test zone mirrored the findings from analyses conducted with our time dependent-variable. In the present experiment, however, age only predicted the proportion of *time spent* within the correct zone, and not the proportion of *distance traveled* within the same zone. Although these two measures were highly correlated across the four experiments we report here, it is of course possible to spend a long period of time in a search zone while traveling a relatively short distance within it.^1^ For example, if a participant was particularly confident in their decision making, they may dwell at the goal location for a relatively long period of time. The observed pattern of data in the current experiment, then, may reflect more confident reorientation decisions in older, compared with younger, children. This then begs the question of why a similar pattern of results was not observed in Experiment 3, for example. One possibility is that the cross-maze environment used in Experiment 4 was the first occasion in our series of experiments in which children were required to reorient during a test trial where both the local- and global-shape cues that were rewarded in training were present, but now placed into behavioral conflict. Thus, a prepotent response (e.g., navigate toward the Local Correct zone) must be inhibited in favor of another response (e.g., navigate toward the Global Correct zone). Perhaps, as children age, decision making under ambiguity is made with greater confidence, and hence *more time specifically* is spent in the Global Correct zone at test.

In addition to demonstrating that children, like adults, reorient on the basis of global-shape representations in the perspective transformation paradigm, the results from the current experiment provide further evidence that children do not reorient on the basis of view-matching. As the exterior of the cross-shaped arena contains the exact local-shape cue that was rewarded during training, view-matching accounts of reorientation, in which participants are thought to navigate by reducing the discrepancy between their current view and a stored snapshot of the view at a rewarded zone of an environment (Cheung et al., 2008; Stürzl et al., 2008; see Cheng et al., 2013, for review), anticipate that participants will preferentially search at the Local Correct corner at test.

## General Discussion

Across four experiments, we aimed to characterize the developmental trajectory of reorientation with respect to global and local representations of boundary shape. Children aged 6–12 years were trained to find a hidden goal with respect to the boundary shape of an environment, before receiving a test trial in which we measured where they searched when the goal was not present. In the shape-transformation paradigm (Experiment 2), children’s ability to reorient on the basis of local-shape cues was examined by transferring them from the inside of a kite-shaped arena to the inside of a rectangle-shaped arena between training and test. Here, age predicted the proportion of time spent searching in the correct zone at test, and above chance performance emerged from the age of 10.5 years. In the perspective transformation paradigm (Experiment 3), children’s ability to reorient on the basis of global-shape cues was examined by transferring them from the inside of a kite-shaped arena to the outside of the same kite-shaped arena between training and test. Again, age predicted the proportion of time spent searching in the correct zone at test, and above chance performance also emerged from the age of 10.5 years. These developmental trajectories did not simply reflect older children being better able to reorient in our virtual paradigm, because when children were trained and tested inside a kite-shaped environment (Experiment 1), age did not significantly predict reorientation ability, and children from the age of six demonstrated above chance performance. Finally, by training participants on the inside of a cross-shaped arena and testing them on the outside of the same arena, we examined children’s preference for reorienting on the basis of global- or local-shape information, as behavior based upon these representations was placed into conflict (Experiment 4). Here, we observed that age predicted the proportion of time spent in the Global Correct zone at test, but not the Local Correct zone, with above chance performance emerging from around 8.5 years.

As noted in the introduction, the extent to which children rely on global or local representations of boundary shape to reorient has been hard to interpret from the extant developmental literature, due to the use of paradigms in which reorientation behavior based on global- and local-shape representations cannot be dissociated. By contrast, studies of reorientation conducted with adult participants have used shape-transformation and perspective- transformation paradigms to isolate reorientation based upon local or global representations of boundary shape, respectively, and it has been demonstrated that adults encode both local- (Buckley et al., 2016a; Lew et al., 2014) and global-shape (Buckley et al., 2016b) representations. By using the same paradigms in Experiments 2 and 3, our data reveal that children’s abilities to reorient using local or global representations of boundary shape develops in parallel and, by around the age of 10.5 years, children display a pattern of performance that is comparable to adults (see Buckley et al., 2016a, 2016b) when reorienting on the basis of either representation of environmental boundaries. This estimate, of course, is based not only on the development of reorientation abilities with age, but also the sensitivity of our tasks and environments to detect successful performance. Indeed, in Experiment 4, children were able to successfully reorient on the basis of a global-shape representation from around the age of 8.5 years, which might be explained by the global shape of the cross-maze in Experiment 4 being more salient than the kite-shape used in Experiment 3, or by the differences in the training protocols between experiments. With this in mind, the observation that children display adult-like global and local reorientation behavior at the age 10.5 years should not be taken as a distinct point of developmental change.^2^ Instead, we wish to highlight what seems to be a more conservative, but yet still surprising, point: when children are recruited and trained in an identical manner, test trials that isolate reorientation based on either local- (Experiment 2) or global-shape (Experiment 3) representations revealed these two forms of competency emerge at the same age, and develop in parallel.^3^

In addition to demonstrating that the ability to reorient on the basis of global- or local-shape cues develops in parallel, we also observed that children in Experiment 4 preferentially reoriented using global-shape representations over local-shape representations, when transferred from the inside to the outside of a cross-shaped arena. As the data from Experiments 2 and 3 demonstrated that children were able to reorient using only local- or only global-shape representations, respectively, the preference observed in Experiment 4 cannot be parsimoniously explained by suggesting that children only encoded the global shape of the cross-maze in Experiment 4. Instead, during training in the cross-maze environment, it is more likely that children encoded the goal location with respect to both global and local representations of the boundary shape. One manner in which biases in search behavior may then be established from multiple representations of boundary shape is via a weighting system that determines which spatial representation is likely to produce the most appropriate behavior, given the current task demands (for a recent instantiation of a weighting model applied to reorientation tasks see Xu et al., 2017; see also Cheng & Newcombe, 2005; Cheng et al., 2007; Nardini et al., 2008; Ratliff & Newcombe, 2008; Twyman & Newcombe, 2010). At test in Experiment 4, then, global-shape representations were (correctly) weighted more heavily than local-shape representations. Similarly, in Experiment 2, it is likely that children encoded both global- and local-shape cues that signaled the goal location, but gave greater weighting to local cues in the test of Experiment 2 because the global shape of the test arena was now different to training, but the local shape cues were the same. Finally, in Experiment 3, having encoded both global- and local-shape representations during training, at test children weighted global cues more than local cues based on the fact the global cues were preserved between training and test, but the local cues were now different.

While the adaptive weighting of cues provides an appealing explanation for the results of Experiments 2 and 3 (in that it is clear that a cue which remains constant throughout the experiment should be weighted more than a cue that appears to change between training and test), the precise mechanism that drives this differential weighting when both cues are available unchanged at test (Experiment 4) will remain a question for future research. One possibility is that local-shape information is strongly associated with the context in which it is acquired (i.e., inside or outside), and is down-weighted following contextual shifts. Consequently, local-shape information may transfer between the inside of two different environments, but not from the inside-to-the-outside of an environment—a suggestion that garners some support from the observations that older children displayed no preference for the Local Correct zone having been transferred from the inside to the outside of a cross-maze (Experiment 4), despite being able to reorient on the basis of local-shape information when transferred from the inside of a kite-shaped arena to the inside of a rectangle-shaped area (Experiment 2). The earlier emergence of reorientation with respect to global-shape in Experiment 4 relative to Experiment 3 may also point to the possibility that children are better able to adaptively weight cues when several possible solutions to a task are presented (Global and Local Correct zones in Experiment 4), compared with circumstances in which the correct solution must be self-generated (recognizing the correct zone based on a global-shape representation in Experiment 3).

Lew et al. (2014) observed successful reorientation behavior in children when both training and testing were conducted inside a rectangle-shaped arena, but not when children were transferred from training in a rectangle to testing in a kite-shaped arena. On the basis of these results, they concluded that younger children (in their case 2- to 4-year-olds) must reorient on the basis of a global representation of boundary shape. The pattern of results from our Experiments 1 and 2 are consistent with those reported by Lew et al. in that younger children (6-year-olds in our sample) showed successful reorientation when training and testing were conducted in the same shaped arena, but not when training and testing took place in kite and rectangle shaped arenas, respectively. However, the results of Experiment 3 suggest that younger children are just as unable to reorient on the basis of global-shape information as they are local shape information. Given this observation, a key question that remains to be answered is how younger (<10.5 years) children in Experiment 1, as well as in the many previous reorientation studies in which training and testing were also conducted inside the same boundary shape (e.g., Hermer & Spelke, 1996), successfully reorient on the basis of the shape of an arena. One possibility is that the shape-based representations encoded by younger children, irrespective of whether they are global or local, are particularly susceptible to the effect of a change between the training and the testing environment. Here, when the environment differs between training and test (Experiments 2 to 4) young children may treat the test environment as completely novel, and subsequently fail to reorient on the basis of any learned information. However, older children may be better able to recognize similarities between the training and test environments and, consequently, reorient on the basis of the local- or global-shape representations that they encoded during training.

In all of the experiments reported here, we explored whether sex differences in reorientation were present in our data. In Experiment 1 and 4, we observed that male participants were faster to locate the goal during training, and in Experiment 1 we also observed that male participants spent more time in the correct zone during test. These observations are consistent with a previous finding in which male participants weighted boundary shape more than landmark cues compared with female participants (Lourenco et al., 2011). We note, however, that we did not observe comparable sex differences in the path-length data in any of our experiments. One possible explanation for these results is that the speed of (or confidence in) reorientation decision making in male participants was greater than in female participants, but both male and female participants were equivalent in their navigational behavior following this decision-making process. We note, however, that this interpretation should be treated with caution, as the sex differences that emerged in two of our experiments were less reliable than the age-related effects that we have observed across all four experiments. It is likely that this reflects the fact that our sample size calculations were designed to detect a reliable effect of age, and that examining sex differences was not the main focus of our research. Consequently, the samples we recruited in every experiment were not sufficiently powered to address the issue of sex differences, and given that small sample sizes can lead to both false positive and false negative effects (see Discussion in Button et al., 2013; Buckley & Bast, 2018), further research will be required to determine whether the sex-related effects we have reported are robust.

We note here that our analyses have focused on time spent and distance traveled within zones at test. While this is consistent with the manner in which we have studied reorientation in adult participants (e.g., Buckley et al., 2016a, 2016b; Buckley, Holden, et al., 2019; Buckley, Smith, & Haselgrove, 2019; Holden et al., 2021), it is a departure from the manner in which other laboratories have measured reorientation processes in animals (e.g., McGregor et al., 2006; Pearce et al., 2004) and children (e.g., Hermer & Spelke, 1994; Learmonth et al., 2008), in which experimenters typically record which corner a participant chooses to search at first (although a response latency measure was included in a developmental study conducted by Smith et al., 2008). We are not aware of any literature that has directly compared the sensitivity of these different dependent measures in studies of reorientation, but it is reassuring that successful reorientation based on local-shape cues in the shape-transformation paradigm has been revealed via analysis of first-choice responses in animals (Pearce et al., 2004), children and adults (Lew et al., 2014), and also via analysis of time spent in zones in studies conducted with animals (Esber et al., 2005), humans (Buckley et al., 2016a), and now children (Experiment 2). Our decision to measure time (or path traversed) in the present studies was motivated by Experiments 3 and 4, as zone analysis is the only appropriate measure for the perspective transformation paradigm when testing is conducted on the outside of a boundary. As we note in the methods of Experiment 3, it is possible to reorient inside of a shape by rotating to bring into view successive walls of the environment. When on the outside of a shape, however, participants can only see a few walls from their starting location, and to reorient must travel (past corners) to bring into view successive boundary walls. As this reorientation process would involve traveling through the zones we used to measure search behavior at test, it is then not possible to determine if a participant’s first choice was deliberate, or simply a behavior that was performed while trying to establish orientation. Similarly, some spatial paradigms record the time at which a participant would have found the goal during test trials, were it present (e.g., Kosaki et al., 2015; Redhead et al., 2013). Again, though, when navigating on the outside of a boundary, it is possible that participants may have entered the goal region while in the process of establishing orientation, rather than through deliberate search.

It is possible that there were some nuances to participant behavior that the current experiments were not sensitive to. For example, observed sex differences could be attributable to differing reorientation strategies between females and males. Similarly, while reorientation according to local or global cues appears to develop in parallel, it is possible that strategies or cue-use might change with age, especially following a context change. Under these circumstances, it may be that the procedures used in the current experiments lack sufficient granularity to distinguish between potential strategies on the basis of exploratory behavior. This is partly due to the aforementioned constraints of negotiating the boundary of a space when exploring its exterior, but also to the relative absence of behavioral constraints when navigating the interior of an environment, during which participants were able to explore freely and, critically, in the absence of a goal on test trials. This makes it difficult to clearly predict and operationalize a set of behaviors that can be reliably associated with one form of strategy or another. However, this is also true for the great majority of published assays of reorientation behavior, where the likelihood of selecting the correct corner (or equivalent location) has remained the core dependent measure for over 30 years. Future studies of these phenomena might, therefore, benefit from considering different ways in which exploratory behavior can be codified and categorized. Methods could draw from allied fields, such as the classification of distributed activity in functional neuroimaging (e.g., Norman et al., 2006) or of scan paths in studies of eye movements (e.g., Cristino et al., 2010). Pellicano et al. (2011) provide an example of the latter approach. They used an algorithm designed for eye movement analysis and revealed that the physical search paths of children exploring a large-scale real-world array were less systematic and less optimal in participants with autism, compared with typically developing counterparts. However, their dependent measures were based on the sequence of inspections of a series of fixed locations, rather than freely exploring an empty space. Making similar changes to reorientation paradigms, that constrain the nature of movements that participants can make, may risk modulating the behavior itself and, therefore, it may not be as straightforward to apply existing analytic techniques to this problem.

More generally, we are mindful that our experiments were conducted using desktop virtual reality, which deprives participants of the idiothetic cues that are present when studying reorientation in a physical laboratory space. While the extent to which self-motion cues are relevant for solving reorientation tasks might be questioned (as participants are disoriented between trials), it is feasible that they may impact upon how children explore the environment, or remember where they have been within a trial (Lourenco & Huttenlocher, 2006). Given that cognitive maps are thought to be built as a function of exploration (O’Keefe & Nadel, 1978), it is then plausible that individual differences in locomotion may predict task success. More pertinently, perhaps, it is also the case that children in our experiments experienced unexpected changes in boundary structure that do not occur in the real world (Experiment 2), and were also transported from the inside to the outside of a boundary (Experiments 3 and 4) without vestibular or proprioceptive feedback. As we noted in the Introduction, these manipulations were necessary to develop paradigms that examine reorientation based solely on global- and local-shape representations. For instance, were participants to be asked to navigate through a doorway in the boundary to traverse from the inside to the outside of the boundary, egocentric updating of spatial information would have confounded our measure of reorientation based on global-shape cues. That is, participants may have kept track of their position with relation to the goal location as they moved across the boundary, and plotted a route to the outside of the corner based on path integration (e.g., the goal is in the corner to my right, so when I exit the building I will turn right). As we have noted earlier, though, developmental studies assessing how children encode boundary representations have successfully used “transportation” procedures (e.g., Negen et al., 2018). Moreover, the procedures we have used are comparable with reorientation studies conducted with rodents, who are placed into experimental apparatuses by researchers (being denied cues that support idiothetic spatial updating), and who also experience unexpected changes between training and test environments (e.g., Pearce et al., 2004). In short, the purpose of our experiments was to track the development of reorientation abilities with respect to boundary shape representations, and the use of virtual environments allowed us to design paradigms in which we could isolate reorientation based on local- or global-shape representations. However, a necessary consequence of these somewhat abstract (relative to real-world behaviors) tasks was to remove alternative reorientation strategies that are likely to be used in everyday navigational behavior.

Consistent with our previous interpretation of behavior in the perspective-transformation paradigm (e.g., Buckley et al., 2016b; Buckley, Holden, et al., 2019; Buckley, Smith, & Haselgrove, 2019), in the present article we have discussed behavior during the test trial of this task as revealing reorientation behavior that is based on a representation of global boundary shape. One objection to this interpretation would be to suggest that, at test in this paradigm, people mentally transform any local shape information that was encoded during training. For instance, people may learn to approach an interior corner where the left-hand wall is longer than a right-hand wall, and when placed on the outside of the shape locate the same corner by mentally flipping these spatial relations. On this account, our perspective-transformation paradigm may be viewed as a form of mental rotation problem, and previous reports have shown these abilities to develop through the ages of 6–11 (e.g., Newcombe, 1989; Piaget & Inhelder, 1948/2013; Snow & Strope, 1990); the same ages as our samples in the present experiment. However, reorientation based only on mental transformations of local-shape cues cannot fully account for the data we have obtained with adult samples using the perspective transformation paradigm. In Experiment 1 of Buckley et al. (2016b), adult participants successfully reoriented on the outside of a kite-shaped arena having been trained to find a goal on the inside of the same shape. In Experiment 2 of Buckley et al. (2016b), participants were again transferred from the inside to the outside of a boundary wall, but the shape arena also changed (e.g., from kite to rectangle, or vice versa). Here, participants displayed no preference for any corner of the test environment. If successful reorientation in Experiment 1 of Buckley et al. was based upon mental transformations of local-shape cues, or some form of spatial perspective taking, and did not rely in any way on global-shape cues, then they should have observed similar results in their Experiment 2. Consequently, while we are unable to definitively rule out explanations of behavior in Experiment 3 which posit some role of mental transformation of local-shape information, it remains the case that, so far, any such strategies have only been detected when the global shape of the training and test environments were congruent—implying that participants are, at some point, using the global shape of the environment during reorientation. Moreover, as Buckley, Smith, and Haselgrove (2019) note in relation to their Experiment 2 (and which applies equally to the current Experiment 4), a mental transformation interpretation would have to assume that a local representation of the goal location elicited via a transformation (i.e., that elicited by the global-correct zone) was better able to control search behavior than a representation that matched the goal location without any mental transformation (i.e., the local-correct zone). It remains to be determined if this assumption is realistic.

A reoccurring theme within the study of navigational behaviors is that some information is afforded a privileged status during learning. Tolman (1948), in his now-classic studies of shortcut taking in rodents, concluded that spatial navigation transcended associative learning between environmental stimuli and rewarded responses, and instead coined the notion of the “cognitive map.” Subsequently, it has been proposed on a number of occasions that allocentric spatial encoding is “modular” in nature, and impervious to interference from nonspatial information–—distinguishing it from other forms of learning (Doeller & Burgess, 2008; see also, Miller & Shettleworth, 2007). In particular, boundary information has been afforded a special status in theories of reorientation and place learning. As we noted earlier, Cheng (1986) suggested that boundary information is processed in an encapsulated module for reorientation, such that learning about boundaries is immune to the interference of landmark cues. Similar conclusions have also been echoed in the place learning literature, where studies in which the presence of landmark cues that have failed to restrict or prevent (or, in the parlance of associative learning, overshadow, or block) learning about boundary information have been taken as evidence of the special status of boundaries in cognitive mapping (Doeller & Burgess, 2008; see also Doeller et al., 2008). There is, then, widespread and reoccurring debate as to whether boundary representations of space are “special,” or whether encoding of space is governed by a more domain-general learning process (e.g., Pearce, 2009). Evidence from our laboratory is beginning to address some of these issues. For example, experiments reported by Buckley, Smith, and Haselgrove (2019) show that global-shape based reorientation following a perspective-transformation is susceptible to interference from nonshape cues in both overshadowing (Pavlov & Anrep, 1927) and blocking (Kamin, 1968) procedures—just like reorientation based on local-shape cues following a shape-transformation (Buckley et al., 2016a). By revealing a comparable developmental trajectory of performance following a shape- and a perspective-transformation, the current experiments join our earlier observations and emphasize a commonality, rather than a distinction, between the two forms of representation used during the reorientation necessitated by these different reorientation paradigms.

### Conclusion

In summary, by adopting shape- and perspective-transformation designs, the experiments presented here characterized the developmental trajectories of reorientation behavior based solely on local- or global-boundary shape information, respectively. Our data suggest that children’s ability to reorient with either source of information develops in parallel, and that global- and local-shape representations are adaptively weighted to determine which controls behavior.

### Context

The study of spatial navigation is a crucible for research activities across many fields in contemporary psychology, with theories that cut across cognitive, comparative, neuroscientific, and developmental domains. A particularly wide-ranging debate, drawing on research from each of the fields above, concerns the way that environmental shape controls reorientation behavior. Here, data from developmental studies have been a particularly important driver of theory development. However, the paradigms used in developmental assays of children’s behavior have often confounded the influence of different environmental cues. During his PhD, Matt Buckley (supervised by Mark Haselgrove and Alastair Smith) developed paradigms that were suitable to revealing the contributions of global- and local-shape representations in guiding reorientation behavior in human adults, which were further refined by Luke Holden during his time as a doctoral student (supervised by Mark Haselgrove, Alastair Smith, and Emma Whitt). The experiments reported here represent our collective efforts in adapting our paradigms for use with a developmental sample, which was collected by the joint first authors of the article. The work forms part of an ongoing collaboration, in which the authors aim to understand the spatial representations that guide our navigational behaviors, and the mechanisms that govern how these representations are encoded.

## Supplementary Material

10.1037/xge0001265.supp

## Figures and Tables

**Table 1 tbl1:** Regression Coefficients From Analyses in Which Age (Months From Youngest) Was Used to Predict Proportion of Time Spent and Distance Traversed in the Correct Zone During the Test Trials of Experiments 1–3

Predicting variable	*B*	95% CI *B*	*SE B*	β	*p*
Proportion of time spent in the correct zone					
Experiment 1					
Constant	.635	[.494, .775]	.071		
Age	.003	[.000, .007]	.002	.221	.072
Experiment 2					
Constant	.367	[.235, .499]	.066		
Age	.004	[.001, .007]	.002	.295	.013
Experiment 3					
Constant	.412	[.284, .539]	.064		
Age	.003	[<.001, .007]	.002	.235	.047
Proportion of distance traveled in correct zone					
Experiment 1					
Constant	.647	[.503, .791]	.072		
Age	.002	[−.001, .006]	.002	.155	.210
Experiment 2					
Constant	.363	[.236, .490]	.064		
Age	.004	[.001, .007]	.002	.280	.018
Experiment 3					
Constant	.420	[.301, .539]	.060		
Age	.003	[<.001, .007]	.002	.250	.034
*Note*. CI = confidence interval.					

**Table 2 tbl2:** Regression Coefficients From Analyses in Which Age (Months From Youngest) Was Used to Predict Proportion of Time Spent and Distance Traversed in the Four Zones Recorded During the Test Trials of Experiments 4

Predicting variable	*B*	95% CI *B*	*SE B*	β	*p*
Proportion of time spent in zone					
Global correct					
Constant	.225	[.129, .322]	.048		
Age	.003	[<.001, .005]	.001	.258	.031
Global incorrect					
Constant	.271	[.182, .359]	.044		
Age	.001	[−.001, .003]	.001	.106	.382
Local correct					
Constant	.215	[.136, .294]	.039		
Age	−.001	[−.003, .001]	.001	−.150	.215
Local incorrect					
Constant	.246	[.168, .324]	.039		
Age	−.002	[−.003, .000]	.001	−.193	.109
Proportion of distance traveled in correct zone					
Global correct					
Constant	.244	[.155, .334]	.045		
Age	.002	[.000, .004]	.001	.190	.115
Global incorrect					
Constant	.279	[.197, .361]	.041		
Age	.001	[−.001, .003]	.001	.113	.352
Local correct					
Constant	.198	[.126, .270]	.036		
Age	−.001	[−.003, .001]	.001	−.103	.395
Local incorrect					
Constant	.236	[.163, .308]	.037		
Age	−.001	[−.003, .001]	.001	−.153	.205
*Note*. CI = confidence interval.					

## Footnotes

1 For Experiments 1–3, there was a strong positive correlation between the proportion of time and distance traversed in the correct zone at test (Experiment 1, *r*(65) = .94, *p* < .001; Experiment 2, *r*(69) = .89, *p* < .001; Experiment 3, *r*(70) = .97, *p* < .001). Time spent and distance traveled within the Global Correct zone were also strongly correlated in Experiment 4, *r*(68) = .95, *p* < .001.

2 On a related note, children in our experiments were naïve to the experimental manipulations, and it is possible that with appropriate scaffolding (e.g., forewarning about changes in boundary-shape and transfers across the boundary between training and test) younger children in our samples may have shown above chance performance. We repeat, though, that the purpose of our experiments was not to highlight a distinct point of developmental change. Instead, we stress that under the same training conditions, test trials that isolate reorientation based on local- and global-shape cues reveal that these abilities develop in parallel.

3 The analyses of test trial performance we present in Experiments 2 and 3 revealed that children, at around the age of 10.5, appeared equally proficient at reorienting on the basis of local and global shape cues, respectively. These analyses, however, are unable to determine if the development of reorientation based on local- and global-shape cues occurs in parallel or is, instead, intersected. To address this issue, following analyses we have reported in previous developmental studies (Buckley et al., 2015; see also Thomas et al., 2009), we compared the slope and intersect of the trajectories observed in Experiments 2 and 3. Here, we performed two univariate ANCOVAs on the proportion of time spent in the correct zone at test, with experiment (2 or 3) as a between-subjects factor, and age as a covariate. For one ANCOVA, the ages of participants were rescaled to reflect the oldest overlapping age in the participants recruited to Experiments 2 and 3, and for the second ANCOVA the ages of participants were rescaled to reflect the youngest overlapping age of both samples. Rescaling the age of participants does not alter the analysis of the age covariate, or the interaction between the age covariate and the between-subjects factor of experiment. It does, however, specify the point at which the main the effect of experiment is calculated. By rescaling age to the oldest overlapping age, a main effect of experiment would indicate a different in performance between Experiments 2 and 3 at the oldest overlapping age in our samples. Similarly, with age rescaled to reflect the youngest overlapping age, a main effect of experiment would indicate a different in performance at the youngest overlapping age in our samples. In keeping with the results we have already reported in Experiments 2 and 3, these ANCOVA analyses revealed a significant age covariate *F*(1, 139) = 10.40, *p* = .002, η_p_^2^ = .07, 95% CI [.02, .15]. The main effect of experiment, however, was not significant when ages were rescaled to reflect the oldest overlapping age in our samples, *F*(1, 139) = .01, *p* = .93, η_p_^2^ < .001, 95% CI [.00, .003], nor when ages were rescaled to reflect the youngest overlapping age, *F*(1, 139) = .13, *p* = .72, η_p_^2^ = .001, 95% CI [.00, .02]. Finally, there was not a significant interaction between the age covariate and experiment, *F*(1, 139) = .07, *p* = .80, η_p_^2^ < .001, 95% CI [.00, .02]. The absence of a main effect of experiment, or an interaction between experiment and the age covariate, suggest that the development of reorientation based on global- and local-shape cues occurs in parallel.
