# Supplementary material for: The Developmental Trajectories of Children’s Reorientation to Global and Local Properties of Environmental Geometry
Source: J Exp Psychol Gen. 2022 Aug 4;153(4):889–912. doi: 10.1037/xge0001265 (PMC11115358; doi:10.1037/xge0001265)
Supplement: Supplementary file 1 [file xge0001265.docx]

| **A** | 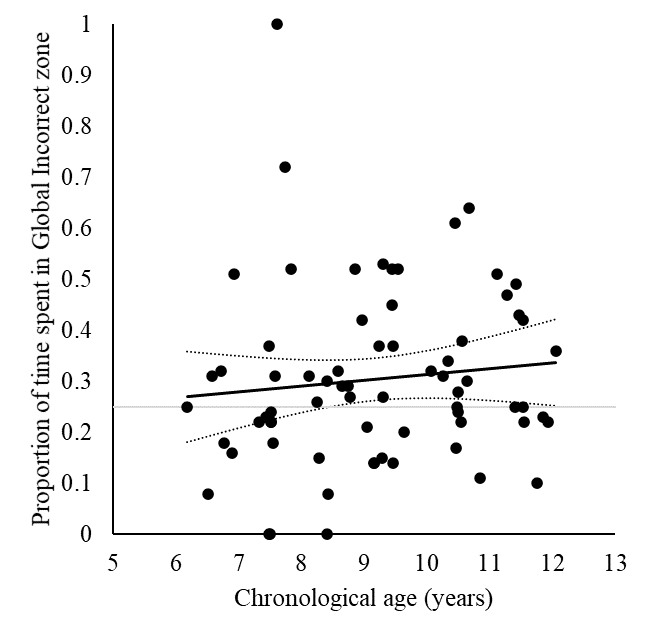 |
| --- | --- |
| **B** | 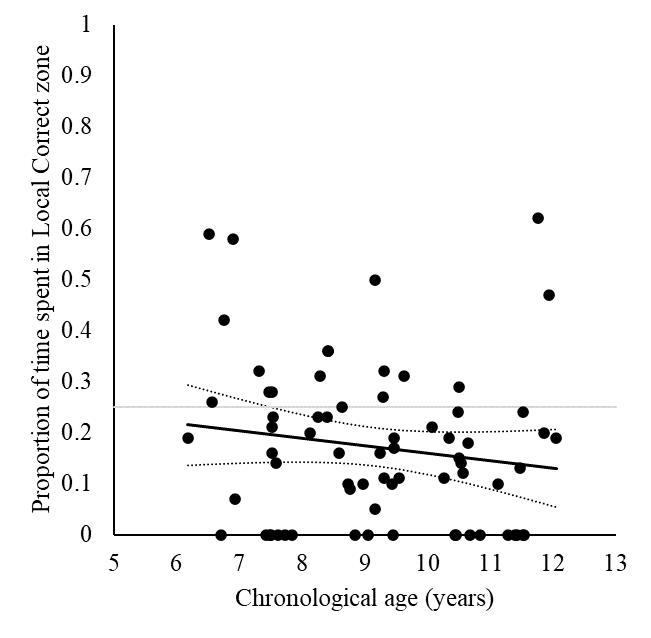 |
| **C** | 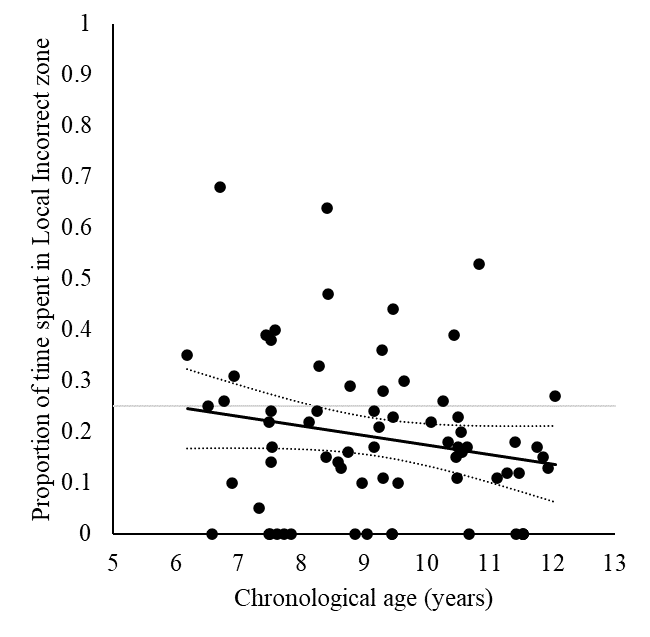 |

**Figure S1.** Proportion of time spent in the Global Incorrect (Panel A), Local Correct (Panel B), and Local Incorrect (Panel C) zones at test, plotted by individual ages. The solid black line represents the linear regression model of age predicting proportion scores, and the dotted lines represent the upper and lower 95% confidence intervals of the model. The solid grey line indicates chance performance at test.
